# Interaction of yeast Rad51 and Rad52 relieves Rad52-mediated inhibition of *de novo* telomere addition

**DOI:** 10.1371/journal.pgen.1008608

**Published:** 2020-02-03

**Authors:** Esther A. Epum, Michael J. Mohan, Nicholas P. Ruppe, Katherine L. Friedman

**Affiliations:** Department of Biological Sciences, Vanderbilt University, Nashville, Tennessee, United States of America; Columbia University, UNITED STATES

## Abstract

DNA double-strand breaks (DSBs) are toxic forms of DNA damage that must be repaired to maintain genome integrity. Telomerase can act upon a DSB to create a *de novo* telomere, a process that interferes with normal repair and creates terminal deletions. We previously identified sequences in *Saccharomyces cerevisiae* (SiRTAs; Sites of Repair-associated Telomere Addition) that undergo unusually high frequencies of *de novo* telomere addition, even when the original chromosome break is several kilobases distal to the eventual site of telomerase action. Association of the single-stranded telomere binding protein Cdc13 with a SiRTA is required to stimulate *de novo* telomere addition. Because extensive resection must occur prior to Cdc13 binding, we utilized these sites to monitor the effect of proteins involved in homologous recombination. We find that telomere addition is significantly reduced in the absence of the Rad51 recombinase, while loss of Rad52, required for Rad51 nucleoprotein filament formation, has no effect. Deletion of *RAD52* suppresses the defect of the *rad51Δ* strain, suggesting that Rad52 inhibits *de novo* telomere addition in the absence of Rad51. The ability of Rad51 to counteract this effect of Rad52 does not require DNA binding by Rad51, but does require interaction between the two proteins, while the inhibitory effect of Rad52 depends on its interaction with Replication Protein A (RPA). Intriguingly, the genetic interactions we report between *RAD51* and *RAD52* are similar to those previously observed in the context of checkpoint adaptation. Forced recruitment of Cdc13 fully restores telomere addition in the absence of Rad51, suggesting that Rad52, through its interaction with RPA-coated single-stranded DNA, inhibits the ability of Cdc13 to bind and stimulate telomere addition. Loss of the Rad51-Rad52 interaction also stimulates a subset of Rad52-dependent microhomology-mediated repair (MHMR) events, consistent with the known ability of Rad51 to prevent single-strand annealing.

## Author summary

DNA double-strand breaks (DSBs) can lead to chromosome loss and rearrangement associated with cancer and genetic disease, so understanding how the cell coordinates multiple possible repair pathways is of critical importance. Telomerase is a ribonucleoprotein enzyme that uses an intrinsic RNA component as a template for the addition of highly repetitive, protective sequences (called telomeres) at normal chromosome ends. Rarely, telomerase acts upon a DSB to create a new or *de novo* telomere with resultant loss of sequences distal to the site of telomere addition. Here, we show that interactions between proteins with known roles during DSB repair modulate the probability of telomerase action at hotspots of *de novo* telomere addition in the yeast genome by influencing the association of Cdc13, a protein required for telomerase recruitment, with sites of telomere addition. Intriguingly, the same interactions that facilitate telomere addition prevent other types of rearrangements in response to chromosome breaks.

## Introduction

The ends of most linear eukaryotic chromosomes are organized into special nucleoprotein structures, telomeres, that are essential for the maintenance of genome stability and integrity. In association with telomere binding proteins, telomeres protect the ends of the chromosomes from being recognized as DNA double-strand breaks (DSBs), thereby preventing inappropriate nucleolytic processing, fusion, and recombination [1]. Telomeres also counteract loss of sequences due to the ‘end-replication problem’ by serving as a primer for telomere synthesis by the specialized ribonucleoprotein enzyme telomerase [2]. The telomerase reverse transcriptase utilizes its intrinsic RNA component as a template for the addition of TG-rich sequence repeats (TG_1-3_ in yeast) to the overhanging 3’ strand at the chromosome terminus [2].

DNA DSBs are among the most toxic forms of DNA lesions, with failed or incorrect repair carrying a high likelihood of sequence loss, rearrangement, and cell death. Therefore, appropriate detection and repair of DSBs is crucial for the maintenance of genome stability. DSBs are generated due to exposure to exogenous agents such as ionizing radiation and radiomimetic drugs or endogenous agents such as reactive oxygen species and replication errors [3]. The two major pathways of DSB repair, homologous recombination (HR) and non-homologous end joining (NHEJ), differ in the requirement for sequence homology. HR utilizes a homologous template such as a homologous chromosome or sister chromatid for repair, while NHEJ involves direct ligation of broken ends with minimal processing [4]. During HR, the 5’ terminating strands are resected to generate 3’ single-stranded DNA that is rapidly bound by replication protein A (RPA), a three-protein complex encoded in yeast by the genes *RFA1-3* [3,5,6]. Association of yeast Rad52 with RPA-coated single-stranded DNA results in the displacement of RPA and formation of the Rad51 nucleoprotein filament [3,7,8] that initiates the homology search and coordinates strand invasion into homologous duplex DNA [3,9]. In addition to serving as a mediator of Rad51 filament formation, Rad52 also facilitates annealing between complementary RPA-coated single strands [10,11].

While NHEJ and HR are the primary pathways of DSB repair in eukaryotic cells, other forms of less accurate repair such as single-strand annealing (SSA) [3], microhomology-mediated end joining (MMEJ) [12], and break-induced replication (BIR) [13] also occur. These ‘non-conservative’ repair pathways lead to formation of gross chromosomal rearrangements (GCRs) including deletions, inversions, and translocations. The genetic requirements for these pathways are different but overlapping, and are influenced by the extent and location of available homologies [14]. Since genome rearrangements influence both cancer and genetic disease through changes in gene dosage, formation of fusion proteins, and/or changes in gene regulation, an understanding of how these non-conservative pathways are regulated and how they may compete during repair of a DSB is essential.

While the pathways described above require interaction of the DSB with an intra- or inter-chromosomal sequence to facilitate repair, terminal deletions can arise through direct addition of a *de novo* telomere by telomerase to an internal DSB [15,16]. In a haploid strain, GCR events within a non-essential terminal region of yeast chromosome 5 are more likely to involve *de novo* telomere addition than any other type of rearrangement, whether they occur spontaneously or in response to a single DSB [17–20]. Sites of *de novo* telomere addition in yeast typically contain at least a single TG-dinucleotide, likely reflecting a requirement for base pairing between the 3’ end of the DSB and the telomerase RNA (which in yeast contains the sequence 5’-CACCACACCCACACAC-3’) [18,19,21]. However, this interaction is insufficient and telomerase recruitment to a DSB occurs through at least two (perhaps non-exclusive) mechanisms. At sites containing very short TG tracts (<4 nt), *de novo* telomere addition depends on interaction between the TLC1 telomerase RNA and yeast Ku70/80 [19], a heterodimeric complex that interacts in a non-sequence specific manner with both telomeres and DSBs. The telomere-binding protein Cdc13 also recruits telomerase to DSBs via its interaction with the telomerase component Est1 [18,22–24]. Cdc13 displays a marked preference for TG-rich, telomere-like sequences [25]. However, even in the absence of obvious TG-rich sequences, chromatin immunoprecipitation (ChIP) experiments reveal association of both Cdc13 and telomerase with regions surrounding a DSB under conditions that encourage the generation of substantial resection (*i*.*e*. when HR is restricted) [26]. The probability of *de novo* telomere addition at TG-repeats of ≤11 nt is decreased by the Mec1-dependent phosphorylation of Cdc13 [18]. DNA damage-dependent phosphorylation of the helicase Pif1 by Mec1 also inhibits telomere addition at DNA breaks [27], although such inhibition is overcome at sites containing at least 34 bp of telomeric sequence in a manner dependent on Cdc13 function [28]. Together, these mechanisms limit frequencies of *de novo* telomere addition at most internal sequences.

We have previously characterized two endogenous hotspots of *de novo* telomere addition on the left arms of yeast chromosomes 5 and 9. These TG-rich sequences, termed SiRTAs (Sites of Repair-associated Telomere Addition), undergo *de novo* telomere addition at frequencies ~200-fold higher than neighboring regions [20], even when the initiating chromosome break is located several kilobases distal to the eventual site of telomere addition [20]. The nomenclature for these sites (SiRTAs 5L-35 and 9L-44) reflects the distance each is located (35kb and 44kb, respectively) from the nearest telomere on that chromosome arm. Both sites display a bipartite structure consisting of a Core sequence within which telomerase acts to initiate *de novo* telomere addition and a Stim sequence that enhances telomere addition at the Core by providing binding site(s) for Cdc13 [20]. The identification of these sequences as hotspots of *de novo* telomere addition provides a tractable system in which to examine the interplay between alternative non-conservative repair pathways.

Telomere addition at SiRTA following a distal chromosome break involves extensive 5’ end resection to expose Cdc13 binding sites in single-stranded DNA and to generate a 3’ terminus that can prime telomere addition by telomerase. Since such extensive single-stranded DNA is expected to form a Rad51 nucleoprotein filament, we investigated roles of the HR-associated proteins Rad51 and Rad52 in *de novo* telomere addition at these SiRTAs. Indeed, previous work showed that recruitment of Cdc13 to DSBs lacking extensive TG repeats is reduced in the absence of Rad51 [26]. Here, we show that Rad51, but not Rad52, is required for normal levels of *de novo* telomere addition at two different SiRTAs after DNA DSB induction. Surprisingly, *de novo* telomere addition is restored in the absence of both proteins, suggesting that Rad51 counteracts an inhibitory effect of Rad52. This activity requires the ability of Rad51 to interact with Rad52, but not its ability to bind single-stranded DNA. Additionally, an allele of *RFA1* that by genetic criteria reduces the interaction between Rfa1 and Rad52 blocks the inhibitory effect of Rad52. The reduction in *de novo* telomere addition in the absence of Rad51 correlates with reduced association of Cdc13 with SiRTA and is rescued by the forced recruitment of Cdc13, suggesting that the association of Rad52 with RPA-bound single-stranded DNA directly or indirectly inhibits Cdc13 binding in a manner relieved by Rad51. In the course of these experiments, we found that the genetic manipulations described above also affect the probability of Rad52-mediated microhomology-mediated repair (MHMR) in the region proximal to SiRTA 9L-44, indicating that direct interactions between Rad51 and Rad52 modulate the relative use of alternative repair pathways in a context-dependent manner.

## Results

### A system for the study of *de novo* telomere addition at SiRTAs

**Fig 1:**
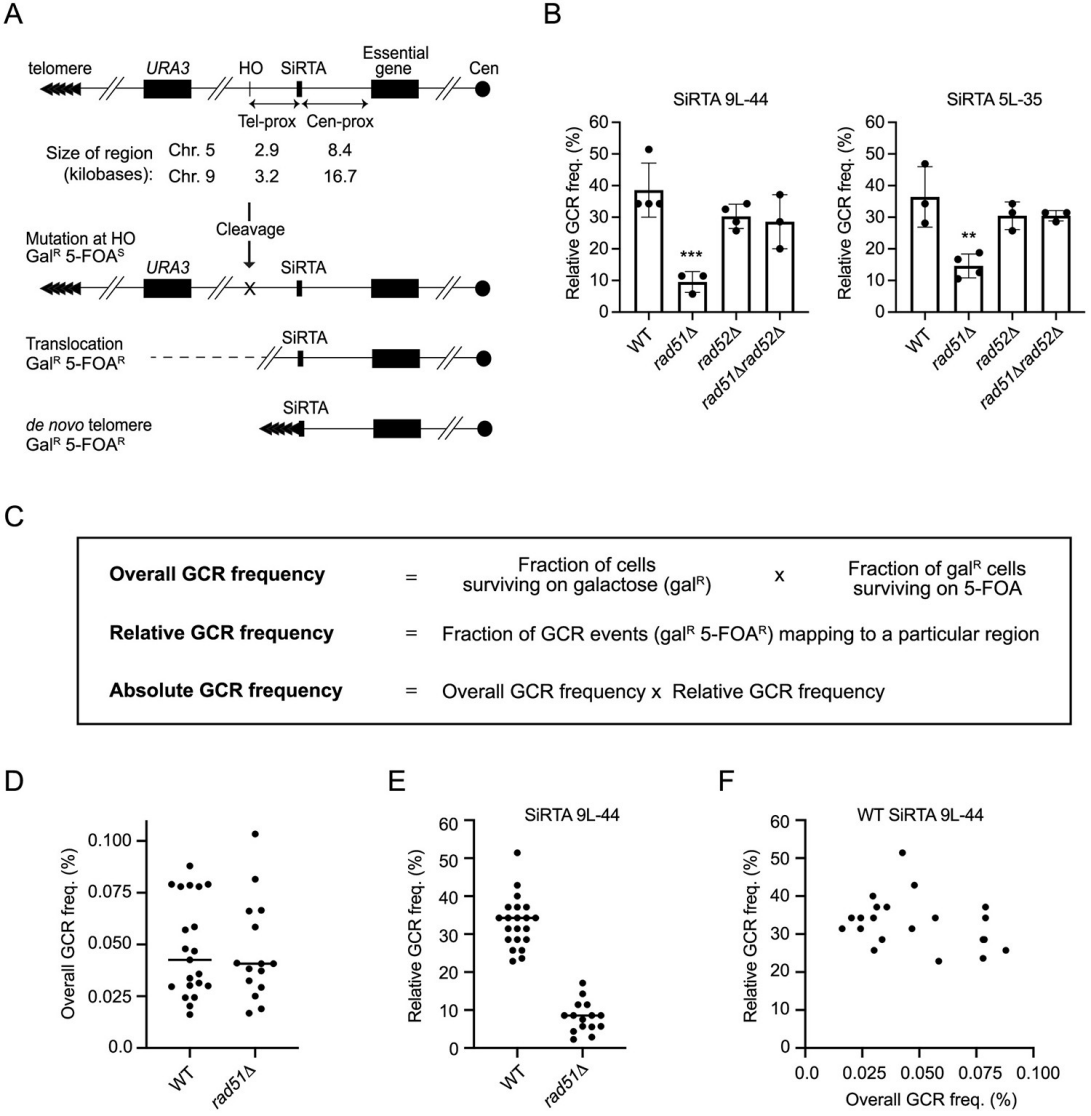
Rad51 promotes *de novo* telomere addition at SiRTAs 9L-44 and 5L-35 by inhibiting Rad52 function. (A) Schematic of the HO cleavage assay system. *MCM10* and *PCM1* are the most distal essential genes on the left arms of chromosome 9 and 5, respectively. Cleavage is induced by the expression of HO endonuclease upon plating on galactose-containing medium, surviving colonies lacking *URA3* function are selected on media containing 5-FOA, and the approximate location of each GCR event is mapped by PCR to the SiRTA, the region centromere-proximal to the SiRTA (Cen-prox), or the region telomere-proximal to the SiRTA (Tel-prox). The sizes in kilobases of each of these regions are shown for the SiRTAs on chromosomes 5 and 9. (B) The relative GCR frequency within the indicated SiRTA is shown for WT and mutant strains. Averages from at least three independent experiments are shown with standard deviations. Strains statistically different from WT by ANOVA with Dunnett’s multiple comparisons test are indicated by asterisks (**p <0.01; ***p <0.001). (C) Definitions and methods for calculating the overall, relative, and absolute frequencies of GCR formation. (D) Overall GCR frequency (%) determined in multiple independent experiments in the *RAD51* (WT) and *rad51Δ* strains after HO cleavage on chromosome 9. Sample numbers are 21 for WT and 15 for *rad51Δ*. (E) Relative GCR frequency in SiRTA 9L-44 from the same experiments shown in D. p<0.0001 by Student’s t test. (F) Plot of overall GCR frequency versus relative GCR frequency in SiRTA 9L-44 for the WT experiments shown in D and E.

The ability of a sequence to function as a SiRTA is monitored in haploid cells using a previously-described inducible HO endonuclease cleavage assay [20,29]. Briefly, a recognition site for the HO endonuclease is integrated approximately 3 kb distal to the SiRTA, while the gene encoding the HO endonuclease is placed under control of a galactose-inducible promoter. Endogenous HO cleavage sites at the *MAT*, *HML*, and *HMR* loci are deleted in this strain background to prevent repair of the break by gene conversion. Finally, the *URA3* gene is integrated approximately 7kb distal to the HO cleavage site to monitor loss of the chromosome end (Fig 1A).

In assays described here, cells are plated on media containing galactose, resulting in persistent HO cleavage. Correct repair (by NHEJ or through use of the sister chromatid as a template during HR) restores the cleavage site and initiates a repair-cleavage cycle that is often lethal; visible colonies form when incorrect repair results in the mutation or removal of the HO recognition site. Loss of the HO site due to a large internal deletion, translocation, or truncation of the chromosome terminus by *de novo* telomere addition causes the cell to acquire 5-fluoroorotic acid (5-FOA) resistance through loss of the distal *URA3* marker (referred to as Gal^R^ 5-FOA^R^ colonies or GCR events) (Fig 1A) [20]. Repair events are constrained between the HO cleavage site and the last essential genes *PCM1* or *MCM10*, located ~43kb and ~60kb from telomeres 5L and 9L, respectively. Sites of repair are subsequently mapped by PCR to three regions: the SiRTA, the region between the SiRTA and the essential gene (Cen-prox), or the region between the SiRTA and the HO site (Tel-prox) (Fig 1A). These regions are not equal in size, with the SiRTA (less than 100bp) encompassing <1% of the distance from the HO site to the essential gene. In previous work, we found that the vast majority of GCR events mapping to SiRTAs 9L-44 and 5L-35 result from *de novo* telomere addition [20] (and see below).

### Rad52 inhibits telomere addition at SiRTA in the absence of Rad51

Because *de novo* telomere addition at SiRTA requires resection from the site of the DSB to expose single-stranded DNA as a template for telomere addition, we examined the role of Rad51, which forms a nucleoprotein filament on single-stranded DNA to facilitate the homology search during HR [9]. Previous work suggested that Rad51 facilitates *de novo* telomere addition, although the mechanism is not understood [26]. Indeed, loss of *RAD51* reduced the fraction of GCR events occurring at SiRTA 9L-44 by 4-fold and at SiRTA 5L-35 by 2.5-fold (Fig 1B and panel A of S1 Fig).

The probability of repair within a particular region can be expressed in two different ways: as a relative GCR frequency (the fraction of all GCR events that occur in that region) or as an absolute GCR frequency (the fraction of all cells that undergo a GCR event in that region). As described in detail below, calculation of the absolute GCR frequency is only advantageous when the overall GCR frequency (the fraction of all cells that give rise to a Gal^R^ 5-FOA^R^ colony) differs significantly between strains. See Fig 1C for definitions and calculations.

On chromosome 9, average overall GCR frequencies in the *RAD51* and *rad51Δ* strains are nearly identical (0.048% versus 0.046%, respectively), but the inherent variability is high (range of 0.072% and 0.086%, respectively; Fig 1D). In contrast, measurements of the relative GCR frequency within SiRTA 9L-44 are more reproducible between experiments and the difference between the *RAD51* and *rad51Δ* strains is highly significant (p<0.0001; Fig 1E). Within a given experiment, there is no correlation between the overall GCR frequency and the relative GCR frequency at SiRTA 9L-44 (Fig 1F). Given this lack of correlation and the variability in the measured overall GCR frequencies, calculating the absolute GCR frequency at SiRTA 9L-44 by multiplying the relative GCR frequency at SiRTA 9L-44 by the overall GCR frequency increases noise without yielding additional information.

Given this analysis, we have adopted the following approach for expressing the data (also see Methods). If the overall GCR frequency of a particular strain does not differ significantly from WT and *rad51Δ*, then results are presented as relative GCR frequencies. When the overall GCR frequency does differ significantly from values measured in the *RAD51* or *rad51Δ* strains, we additionally present the results as absolute GCR frequencies.

If formation of the Rad51 nucleoprotein filament contributes to telomere addition at SiRTAs, then loss of Rad52 (required for filament formation [7,8]) should have the same effect. Notably, deletion of *RAD52* does not decrease the relative GCR frequency at either SiRTA 9L-44 or 5L-35 (Fig 1B and panel A in S1 Fig), consistent with a previous report [20]. In fact, because the overall GCR frequency is modestly higher in the *rad52Δ* strain, the absolute GCR frequency is increased at SiRTA 5L-35 (panel B in S1 Fig). The effect of deleting *RAD51* is unique among the genes of the *RAD52* epistasis group: deleting *RAD54*, *RAD55*, *RAD57* or *RAD59* does not significantly reduce the relative frequency of GCR formation at either SiRTA (panel A in S2 Fig).

We then examined the epistatic relationship between *RAD51* and *RAD52*. Surprisingly, additional deletion of *RAD52* fully suppresses the reduced GCR formation observed in the *rad51Δ* strain at both SiRTA 9L-44 and SiRTA 5L-35 (Fig 1B and S1 Fig). This epistatic relationship is incompatible with a model in which Rad51 directly promotes *de novo* telomere addition at SiRTAs and instead suggests that Rad52 inhibits telomere addition, but only in the absence of Rad51. Importantly, the effects observed at SiRTAs are not due to impaired telomerase activity *per se*, as endogenous telomere length is unaffected by the deletions of *RAD51* and/or *RAD52* (S3 Fig). We conclude that the effects reported here are specific to the disruption of Rad51 function, with Rad52 functioning as a downstream effector.

To examine the type of repair occurring at the SiRTA, a subset of GCR events mapping by PCR to the SiRTA were analyzed by Southern blot (S4 Fig). In a control strain (never exposed to galactose), cleavage of genomic DNA with *Nsi*I yielded the expected band of ~8 kb when a probe immediately internal to SiRTA 9L-44 was utilized (panel A and lane 19 in panel B of S4 Fig). In contrast, the majority of strains with a GCR event mapping to SiRTA 9L-44 yielded a smeary band of ~2.8 kb (panel B in S4 Fig). Since the *Nsi*I site lies ~2.5 kb internal to SiRTA 9L-44, this is the size expected following addition of ~250–350 bp telomeric DNA. These same fragments were detected when the blot was reprobed with a cloned yeast telomeric sequence (panel C in S4 Fig). Strains lacking the signal indicative of telomere addition at SiRTA each showed a single discrete band of varying sizes, indicative of a translocation or large deletion.

Using this approach, 46 of 50 GCR events (92%) mapping to SiRTA 9L-44 in the WT strain were found to involve *de novo* telomere addition. Similar results were observed in cells lacking *RAD52* only or both *RAD51* and *RAD52* [39 of 41 (95%) in *rad52Δ* and 29 of 29 in *rad51Δ rad52Δ*]. In contrast, only 10 of 19 (53%) events mapping to SiRTA 9L-44 in *rad51Δ* cells involved *de novo* telomere addition (S1 Table). We have previously observed that *cis*-acting mutations reducing SiRTA function can reduce the proportion of repair events in the SiRTA that involve *de novo* telomere addition [20], consistent with the effect observed upon *RAD51* deletion. However, the probability of translocation/deletion may be context-dependent, since the fraction of GCR events involving telomere addition in the *rad51Δ* strain was not reduced at SiRTA 5L-35 (S1 Table). We conclude that the vast majority of GCR events at SiRTAs involve *de novo* telomere addition, but that, in the absence of *RAD51*, the frequency of *de novo* telomere addition at SiRTA 9L-44 is even further reduced from that estimated based on GCR frequency alone.

### Rad51 inhibits Rad52-dependent repair events centromere-proximal to SiRTA 9L-44

**Fig 2:**
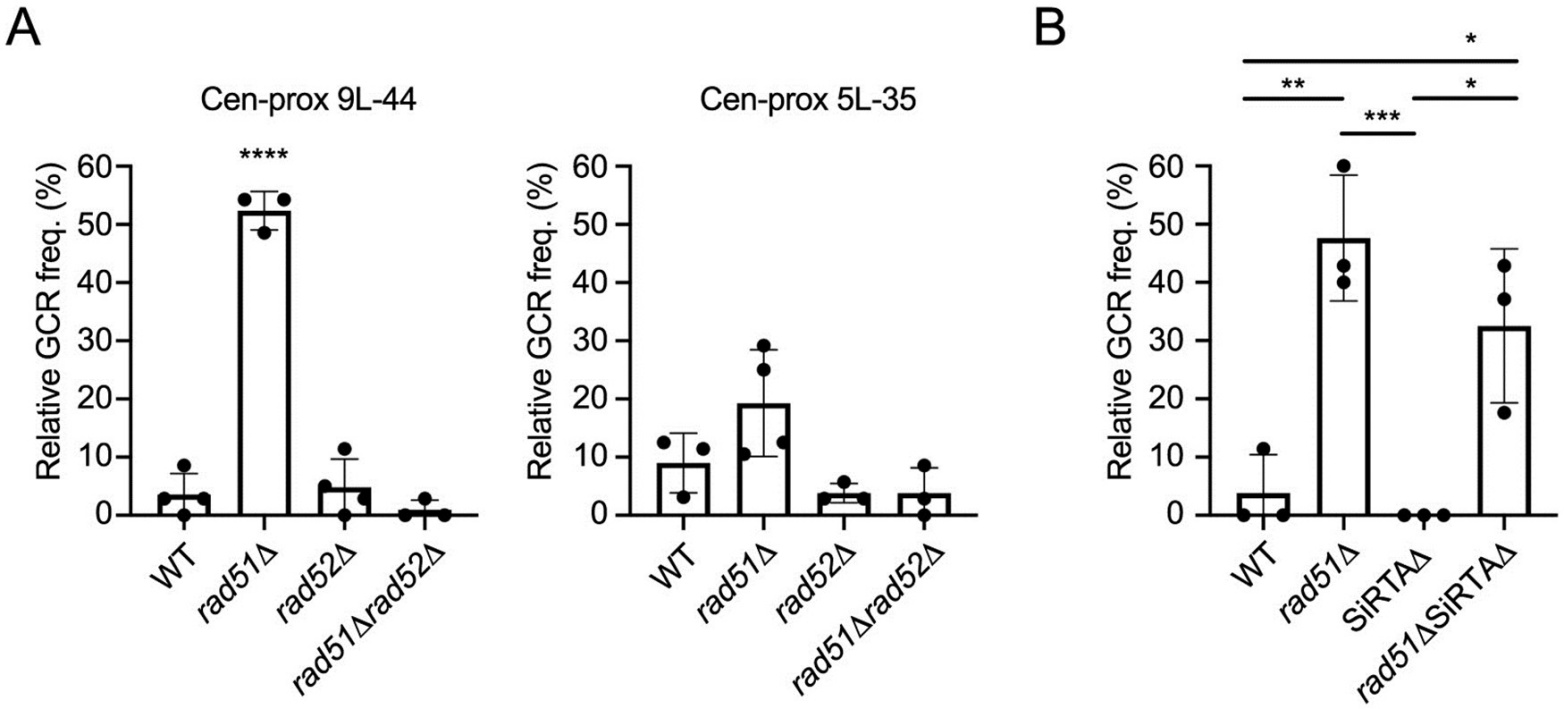
A subset of repair events centromere-proximal to SiRTA 9L-44 increases in frequency upon deletion of *RAD51*. (A) The relative GCR frequency in the region centromere-proximal to SiRTAs 9L-44 and 5L-35 is shown for the indicated strains. The strain statistically different from WT by ANOVA with Dunnett’s multiple comparisons test is indicated by asterisks. (B) The relative GCR frequency in the region centromere-proximal to SiRTA 9L-44 is shown for the indicated strains. In the SiRTAΔ strain, no GCR events were observed in the centromere-proximal region. Values are averages from three independent experiments; error bars represent standard deviation. Averages indicated by asterisks are statistically different by ANOVA with Tukey’s multiple comparisons test (*p<0.05; **p <0.01; ***p<0.001; ****p<0.0001).

During our analysis of telomere addition at SiRTAs, we noticed that the relative frequency of centromere-proximal events is elevated in the absence of *RAD51*. This effect is particularly pronounced within the region centromere-proximal of SiRTA 9L-44 and is suppressed by deletion of *RAD52* (Fig 2A and S1 Fig). As a result, despite the striking change upon deletion of *RAD51*, the distribution of GCR events across all three regions (centromere-proximal, SiRTA, and telomere-proximal) is similar to WT in both *rad52Δ* and *rad51Δ rad52Δ* strains (S1 Fig). At SiRTA 5L-35, there is a trend toward increased relative GCR frequency in the centromere-proximal region, but the difference is not statistically significant compared to WT (Fig 2A and S1 Fig). We do not fully understand the discrepancy between the two SiRTAs, but speculate that differences in the sequence and/or size of the centromere-proximal regions may play a role (the centromere proximal region on chromosome 9 is twice the size of the corresponding region on chromosome 5; Fig 1A).

We wondered whether the reduced telomere addition at SiRTA 9L-44 in the absence of *RAD51* might directly cause the increase in centromere-proximal events by allowing resection to proceed internally in a higher fraction of cells. If true, cells completely lacking SiRTA sequences (SiRTAΔ) should undergo more events in the centromere-proximal region. Instead, we found no centromere-proximal GCR events in SiRTAΔ 9L-44 cells (Fig 2B). However, such events are not dependent on the SiRTA, since centromere-proximal events are observed in SiRTAΔ *rad51Δ* cells (Fig 2B). We conclude that the phenomena observed at the SiRTA and centromere-proximal regions are independent.

### Rad51-inhibited repair events require Rad59 and Pol32

**Fig 3:**
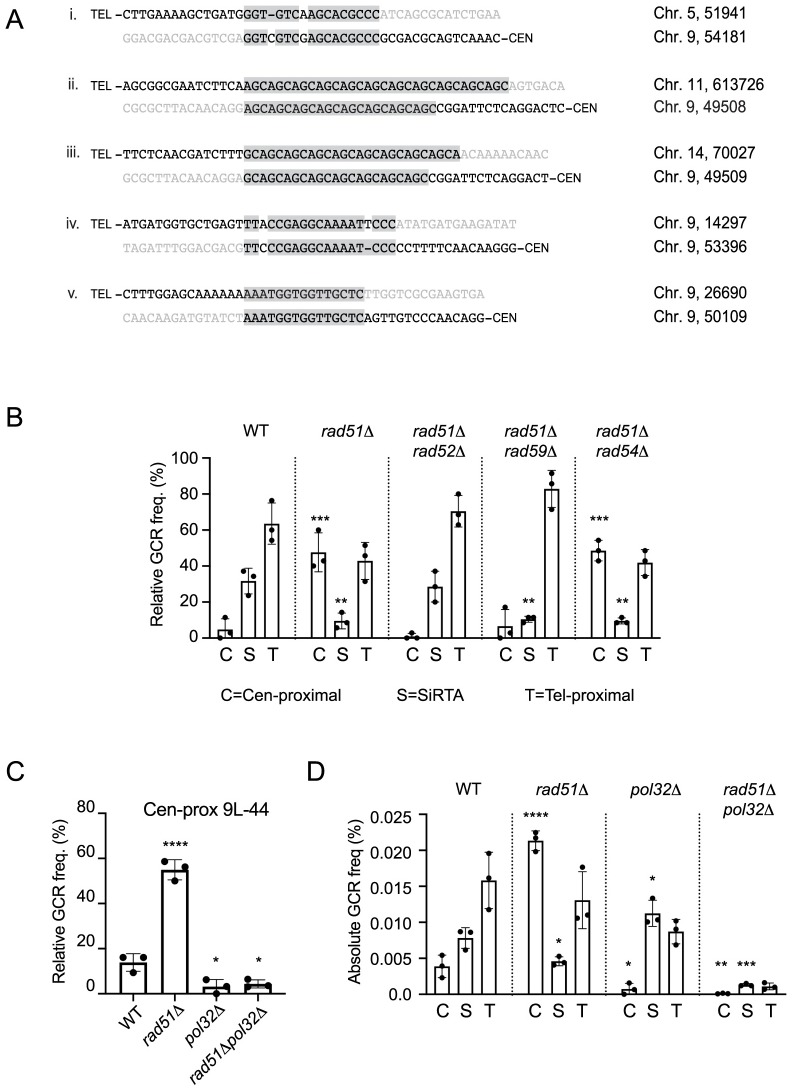
Microhomology-mediated repair requires Rad52, Pol32, and Rad59 and is inhibited by Rad51. (A) Unique breakpoint sequences identified by nanopore sequencing in twelve independent *rad51Δ* strains are shown. Event (iv) was recovered independently eight times, while the other rearrangements occurred once. Bases in gray are present on the original chromosome, while bases in black are those retained in the rearranged chromosome. The shaded regions indicate microhomologies utilized in mediating repair. The chromosome coordinate of each rearrangement is indicated. Additional information is available in S1 Data. (B) The relative GCR frequency in each region on chromosome 9 is shown in the indicated strains. C, S, and T indicate centromere-proximal, SiRTA, and telomere-proximal events, respectively. Data for *rad51Δ rad52Δ* are repeated from Figs 1B and 2A for comparison. Values are averages from 3 independent experiments with standard deviation. For the centromere-proximal and SiRTA regions only, averages were compared to the WT sample in that same region by ANOVA with Dunnett’s multiple comparisons test. (C) The relative GCR frequency in the region centromere-proximal to SiRTA 9L-44 is shown for the indicated strains. Averages indicated by asterisks are statistically different by ANOVA with Dunnett’s multiple comparisons test. (D) The absolute GCR frequency (see Fig 1C for calculation) in each region on chromosome 9 is shown in the indicated strains from the same experiments as panel C. Values are averages from 3 independent experiments with standard deviation. For the centromere-proximal and SiRTA regions only, averages were compared to the WT sample in that same region by ANOVA with Dunnett’s multiple comparisons test (*p<0.05; **p <0.01; ***p<0.001; ****p<0.0001).

To gain insight into the types of repair events occurring in the centromere-proximal region, genomic DNA was analyzed from twelve independent *rad51Δ* strains containing breakpoints that mapped between SiRTA 9L-44 and the last essential gene. We used nanopore sequencing at low coverage to obtain very long (up to 54 kb) sequence reads (see Methods). In each case, at least one (and in most cases, multiple) reads spanned the breakpoint, allowing identification of the sequences involved. Each rearrangement was subsequently verified by PCR amplification. As shown in Fig 3A (repair junctions i-iii), three of the strains contain translocations between the left arm of chromosome 9 and chromosome arms 5L, 11R, or 14L, respectively. These strains survived cleavage of chromosome 9 by acquiring ~50–70 kilobases of terminal sequence, including the telomere, from a non-homologous chromosome. Sequence reads from the intact chromosome (5, 11, or 14) are also present in the dataset, indicating that the translocations are nonreciprocal. Microhomology is evident at each breakpoint (Fig 3A), with two of the translocations (to 11 and 14) involving the same trinucleotide repeat on chromosome 9. All three translocations occurred after resection of 10 kb or more from the HO cleavage site, which is inserted ~41.5 kb from the left telomere of chromosome 9.

One additional strain contains a 23 kb deletion on chromosome 9 (Fig 3A, repair junction v), while at least four, and likely all eight, of the remaining strains contain an identical 39 kb internal deletion (Fig 3A, repair junction iv). There is some ambiguity in the latter case because the left termini of chromosomes 9 and 10 are nearly identical over more than 15 kb, including the position of the distal breakpoint [30]. A single base polymorphism between the two chromosomes at nucleotide position 8415 (chromosome 9) could be used in four strains to determine unambiguously that the rearrangement is a deletion, rather than a non-reciprocal translocation to chromosome 10. Appropriate sequence reads were lacking in the remaining four strains to make a determination, but we consider it likely that these are also internal deletions on chromosome 9. Like the translocations, the deletions occur at regions of microhomology (Fig 3A). Using primers designed to amplify across the breakpoint of the common 39 kb deletion (S1 Data), we found that 45% of all centromere-proximal GCR events in the *rad51Δ* background are of this type (30 of 67), while the same deletion accounted for only one of 45 centromere-proximal events (~2%) in the wild-type strain. We conclude that much of the increase in centromere-proximal events upon loss of *RAD51* is driven by an increase in the likelihood of an internal deletion.

The inhibition of centromere-proximal events is unique to Rad51 as individual deletion of *RAD54*, *RAD55*, *RAD57* or *RAD59* resulted in little or no increase in the relative GCR frequency centromere-proximal to SiRTAs 9L-44 or 5L-35 (panel B of S2 Fig). However, as described above, the increase in centromere-proximal events in the *rad51Δ* strain requires *RAD52*, consistent with a homology-driven repair process (Fig 2A and S1 Fig). We next tested the role of *RAD59*, which is required for some Rad51-independent homologous repair pathways [31–34]. Indeed, deletion of *RAD59* suppressed the increase in centromere-proximal events observed in the absence of *RAD51*, while deletion of *RAD54* had no effect (Fig 3B). Importantly, the reduced relative GCR frequency at SiRTA 9L-44 was not suppressed in the *rad59Δ* strain (Fig 3B), further supporting our conclusion that events occurring in the centromere-proximal region are independent of SiRTA function.

We also examined the role of *POL32*, which encodes a nonessential subunit of DNA polymerase δ [35] and is required for some repair events requiring extensive replication [36,37]. Even in a WT *RAD51* background, the relative and absolute GCR frequencies in the centromere-proximal region are reduced upon deletion of *POL32* and this phenotype is epistatic to *rad51Δ* (Fig 3C and 3D). When both *RAD51* and *POL32* are deleted, the absolute frequency of repair in all regions is markedly decreased (Fig 3D). We conclude that nearly all of the centromere-proximal events, in both the presence and the absence of *RAD51*, require *POL32*.

### The negative effect of Rad52 on *de novo* telomere addition requires interaction with Rad51

**Fig 4:**
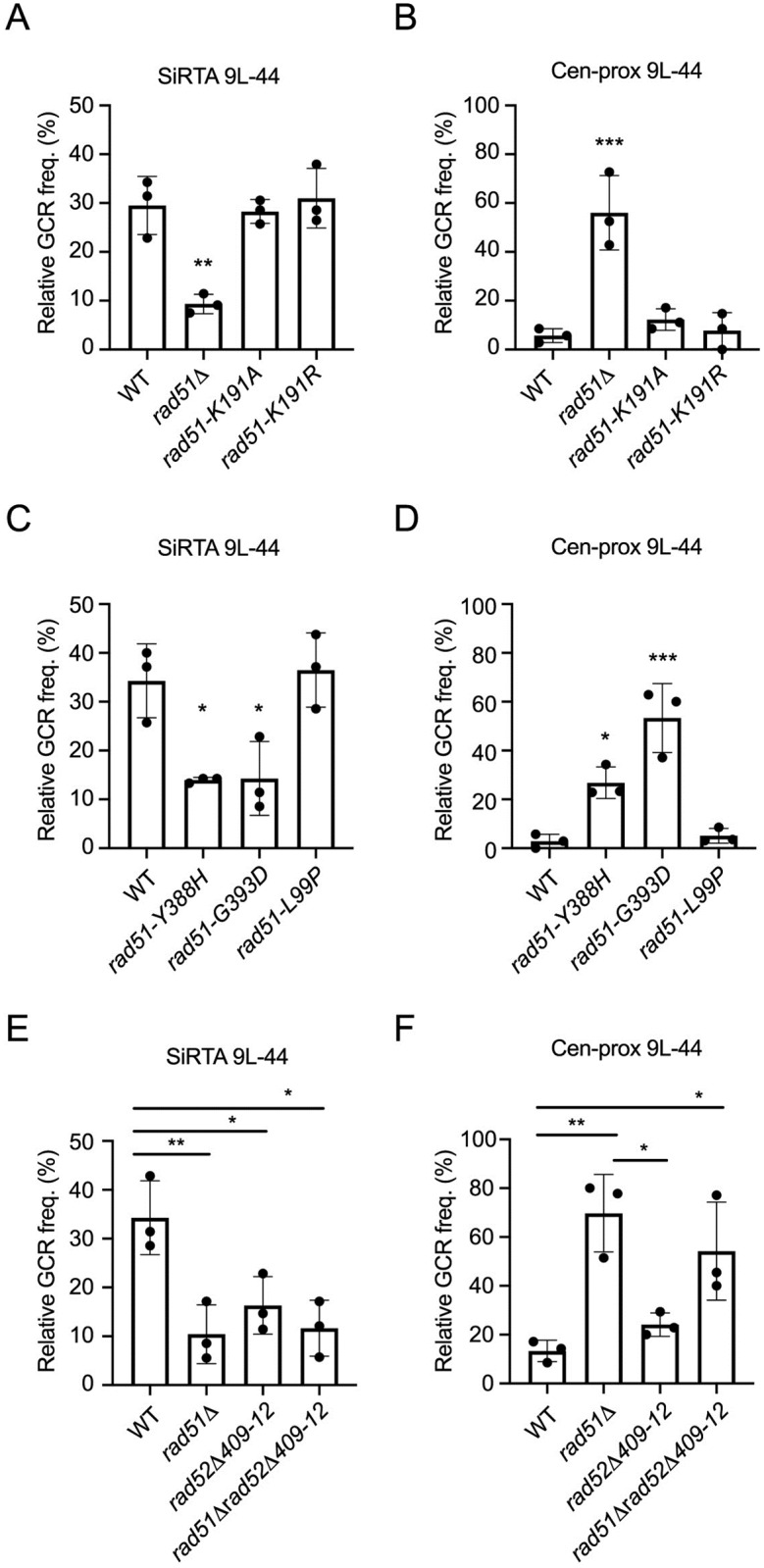
The Rad52-dependent effects of Rad51 on telomere addition and micro-homology mediated repair require the Rad51-Rad52 interaction. (A-D) The relative GCR frequency in SiRTA 9L-44 (A, C) or the centromere-proximal region (B, D) is shown for the indicated strains. Averages and standard deviations are from three independent experiments. Strains statistically different from the corresponding WT control strain by ANOVA with Dunnett’s multiple comparisons test are indicated by asterisks. (E-F) The relative GCR frequency in SiRTA 9L-44 (E) or the centromere-proximal region (F) is shown for the indicated strains. Averages and standard deviations are from three independent experiments. Values that are statistically different by ANOVA with Tukey’s multiple comparison test are indicated by asterisks (*p<0.05, **p<0.01; ***p<0.001). Overall GCR frequencies of the strains analyzed in this figure did not differ significantly from those frequencies measured in the *RAD51* and *rad51Δ* strains.

The incongruence between the effects of *RAD51* and *RAD52* deletion on *de novo* telomere addition at SiRTAs implies that the requirement for Rad51 is independent of nucleoprotein filament formation. To test this idea, we assayed strains expressing *rad51* alleles that block ATP binding (K191A) or hydrolysis (K191R) by Rad51, activities required for normal formation of the Rad51 nucleofilament [38,39]. Both alleles were integrated at the endogenous *RAD51* locus. By Western blot, these protein variants are expressed at levels only slightly reduced compared to WT (S5 Fig). Importantly, despite profound defects in single-stranded DNA binding (K191A) and defective nucleoprotein filament formation (K191R), both proteins retain association with Rad52 as measured by co-immunoprecipitation, albeit at reduced levels (S5 Fig). The retention of Rad52 interaction by Rad51-K191R is in agreement with a previous report [40]. Both alleles support normal levels of GCR formation at SiRTA 9L-44 (Fig 4A) and do not show the markedly increased relative frequency of GCR events in the centromere-proximal region that is characteristic of the *rad51Δ* strain (Fig 4B). Importantly, although the Rad51-K191A variant is unable to bind single-stranded DNA or form nucleofilament, 25 of 25 GCR events mapping to SiRTA 9L-44 are *de novo* telomere addition events, again consistent with this variant retaining function in this assay (S1 Table). In the *rad51-K191R* strain, 19 of 25 events (76%) mapping to the SiRTA involve *de novo* telomere addition (S1 Table).

Given these results, we speculated that physical association between Rad51 and Rad52 might be required to prevent Rad52 from inhibiting *de novo* telomere addition at SiRTAs. To test this hypothesis, we analyzed cells expressing the Rad51 variants Y388H and G393D, shown by yeast two-hybrid and biochemical experiments to be defective for interaction with Rad52 (but not with Rad54 or Rad55) [41,42]. We confirmed that the rad51-Y388H and rad51-G393D proteins are expressed at levels equivalent to WT when integrated at the endogenous locus, but do not co-immunoprecipitate with Rad52 (S5 Fig). As predicted, both the *rad51-Y388H* and *rad51-G393D* strains show reduced GCR formation at SiRTA 9L-44 (Fig 4C) and increased relative GCR frequency in the centromere-proximal region (Fig 4D) compared to a strain expressing WT *RAD51*. Finally, to confirm that other members of the *RAD52* epistasis group are not required, we tested the effect of the *rad51-L99P* allele. The L99P mutation disrupts interaction of Rad51 with Rad54 and Rad55 [41], but the interaction with Rad52 is retained (S5 Fig). The relative GCR frequency at both SiRTA 9L-44 and the centromere-proximal region in the *rad51-L99P* strain is indistinguishable from WT (Fig 4C and 4D). Consistent with this phenotype, 24 of 25 events mapping to SiRTA 9L-44 in the *rad51-L99P* strain involve *de novo* telomere addition (S1 Table).

If interaction between the two proteins is critical, we would expect a mutation in Rad52 that disrupts interaction with Rad51 to reduce GCR formation at SiRTA, regardless of the status of Rad51 (*i*.*e*. Rad51 would be incapable of blocking the negative effect of the Rad52 variant on *de novo* telomere addition). We examined the effect of deleting Rad52 residues 409 to 412, a mutation that disrupts DNA repair and the association of Rad51 with Rad52 (thereby eliminating Rad52 mediator activity), but does not affect DNA binding, ssDNA annealing, or protein oligomerization by Rad52 [43]. Integration of the *rad52Δ409–412* allele at the *RAD52* locus reduced the relative frequency of GCR formation at SiRTA 9L-44 to an extent similar to deletion of *RAD51* (Fig 4E). Furthermore, combining this *rad52* allele with a deletion of *RAD51* neither suppressed nor further reduced the relative frequency of GCR events at SiRTA 9L-44 (Fig 4E). Consistent with this observed reduction in relative GCR frequency at SiRTA 9L-44 in the strains expressing *rad52Δ409–412*, we observed a decline in the fraction of SiRTA events involving *de novo* telomere addition (75% in *rad52Δ409–412* and 66.67% in *rad51Δ rad52Δ409–412*; S1 Table). Taken together, these results suggest that it is not formation of the nucleoprotein filament or strand exchange by Rad51 *per se* that are required for *de novo* telomere addition. Rather, Rad51 must interact with Rad52 to block the ability of Rad52 to inhibit *de novo* telomere addition.

As described above, we see a strong correlation between those alleles that reduce GCR formation at SiRTA and those that increase GCR formation in the centromere-proximal region. The exception to this rule is *rad52-Δ409–412*, which does not significantly increase the relative frequency of events in the centromere-proximal region (Fig 4F). Since our previous results argue that effects at SiRTA and the centromere-proximal region can occur independently, we conclude that the interaction between Rad51 and Rad52 serves to suppress formation of microhomology-mediated rearrangements, although there is some context dependence to this effect since we do not observe a statistically significant increase in the corresponding region of chromosome 5 (Fig 2A and S1 Fig).

### Defective checkpoint adaptation does not obligately reduce *de novo* telomere addition at SiRTA

**Fig 5:**
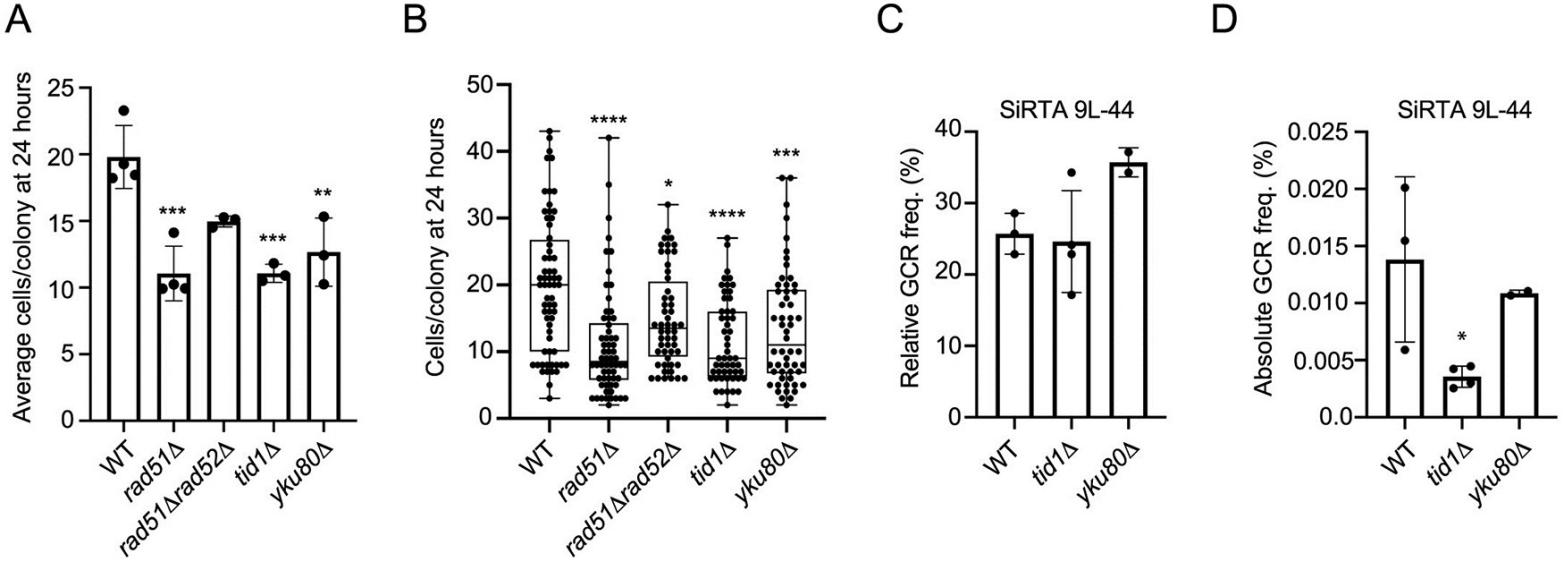
Adaptation-defective strains do not show consistently reduced GCR formation at SiRTA. Individual unbudded cells from the indicated strains were micro-manipulated on an agar plate containing galactose (to induce HO endonuclease expression). After 24 hours, the number of cells in each colony was determined. (A) The average number of cells per colony after 24 hours is plotted as an average and standard deviation of 3 or 4 independent experiments. Each experiment followed the growth of 11–18 micromanipulated cells. Values that are statistically different from WT by ANOVA with Tukey’s multiple comparisons test are indicated by asterisks. All other pairwise comparisons are not significant. (B) Box and whisker plots show the median and 25 and 75% range of colony size for all micromanipulated cells analyzed in experiments summarized in A. Values that are statistically different from WT by ANOVA with Tukey’s multiple comparisons test are indicated by asterisks. All other pairwise comparisons are not significant. (C) The relative GCR frequency in SiRTA 9L-44 is shown for the indicated strains. (D) The absolute GCR frequency in SiRTA 9L-44 is shown for the indicated strains from the same experiment shown in C. For C and D, averages and standard deviations are from at least two independent experiments. Strains statistically different from the corresponding WT control strain by ANOVA with Dunnett’s multiple comparisons test are indicated by asterisks (*p <0.05; **p<0.01; ***p<0.001; ****p<0.0001).

The genetic results presented above suggest that Rad52 can interfere with *de novo* telomere addition at SiRTA, but that this effect is alleviated through the Rad52-Rad51 interaction. Haber and colleagues reported the same genetic interaction between *RAD52* and *RAD51* in the context of checkpoint adaptation [39]. Yeast cells subjected to a persistent DSB arrest in G2/M, but eventually release from the checkpoint and proceed into the following cell cycle, even in the absence of repair [39]. Cells lacking *RAD51* have a moderate adaptation defect that is suppressed by deletion of *RAD52* and, as we observe for *de novo* telomere addition, suppression requires the Rad52-Rad51 interaction and is independent of Rad51-nucleoprotein filament formation [39]. We tested the adaptation phenotype of the WT, *rad51Δ*, and *rad51Δ rad52Δ* strains undergoing HO-induced cleavage on chromosome 9 by micro-manipulating single unbudded cells immediately after transfer to plates containing galactose and counting the number of cells per microcolony after 24 hours. We observed a significant reduction in the average colony size in the *rad51Δ* strain compared to WT and this difference was partially rescued in the *rad51Δ rad52Δ* strain (Fig 5A and 5B). These differences were observed when the average colony sizes from 3–4 independent experiments were compared between strains (Fig 5A) and when the results of these independent experiments were combined (Fig 5B). Only 3.1% of the WT and 11.5% of the *rad51Δ rad52Δ* colonies contained 6 or fewer cells after 24 hours (p = 0.14), while 32.3% of the *rad51Δ* colonies contained 6 or fewer cells (p<0.0001 compared to WT, Fisher’s exact test). We conclude that cells lacking *RAD51* display an adaptation defect that is rescued by additional deletion of *RAD52*, although the magnitude of the adaptation defect is smaller than reported by the Haber lab in a similar experiment (with HO cleavage on chromosome 3) [39]. As expected from the low survival of all strains in response to persistent HO cleavage, none of the micro-manipulated cells gave rise to colonies visible by eye.

Despite the similarities in the genetic interactions, the effects that we observe on *de novo* telomere addition do not appear to be an indirect consequence of the adaptation defect of the *RAD51* deficient strain. Strains lacking either the Ku complex or Tid1 are also adaptation-defective through mechanisms distinct from that occurring in the absence of Rad51 [44,45]. We recapitulate this observation in our system, with strains lacking *TID1* or *YKU80* showing a significantly reduced average colony size compared to WT (Fig 5A and 5B) and 25.0% and 24.1% of colonies containing 6 or fewer cells after 24 hours, respectively (p = 0.0006 and p = 0.0007 compared to WT by Fisher’s exact test; below the Bonferroni corrected α-value of p = 0.0125). However, despite the adaptation defect of these strains, we observe no significant reduction in the relative GCR frequency at SiRTA 9L-44 in either *tid1Δ* or *yku80Δ* strains (Fig 5C), although we note that the absolute GCR frequency is decreased in the *tid1Δ* strain due to a significant reduction in the overall GCR frequency in that strain (Fig 5D). Given the strong parallels in the genetic observations between these two phenomena, it is possible that the underlying mechanisms giving rise to both the *de novo* telomere addition defect and the adaptation defect in the absence of *RAD51* are similar.

### In the absence of Rad51, Rad52 reduces recruitment of Cdc13 to SiRTA following a DSB

Both chromatin immunoprecipitation (ChIP) and immunofluorescence studies show that the association of Cdc13 with DNA (following HO- or chemically-induced DSBs) is stimulated by Rad51 [26,46]. We previously observed that high levels of *de novo* telomere addition at SiRTAs correlates with the ability of the SiRTA-stim sequence to bind Cdc13 and that the effect of the stim sequence is mimicked by artificial recruitment of Cdc13 [20]. These observations suggest that reduced *de novo* telomere addition in the absence of *RAD51* likely reflects reduced recruitment of Cdc13 to SiRTA sequences following an HO-induced DSB.

**Fig 6:**
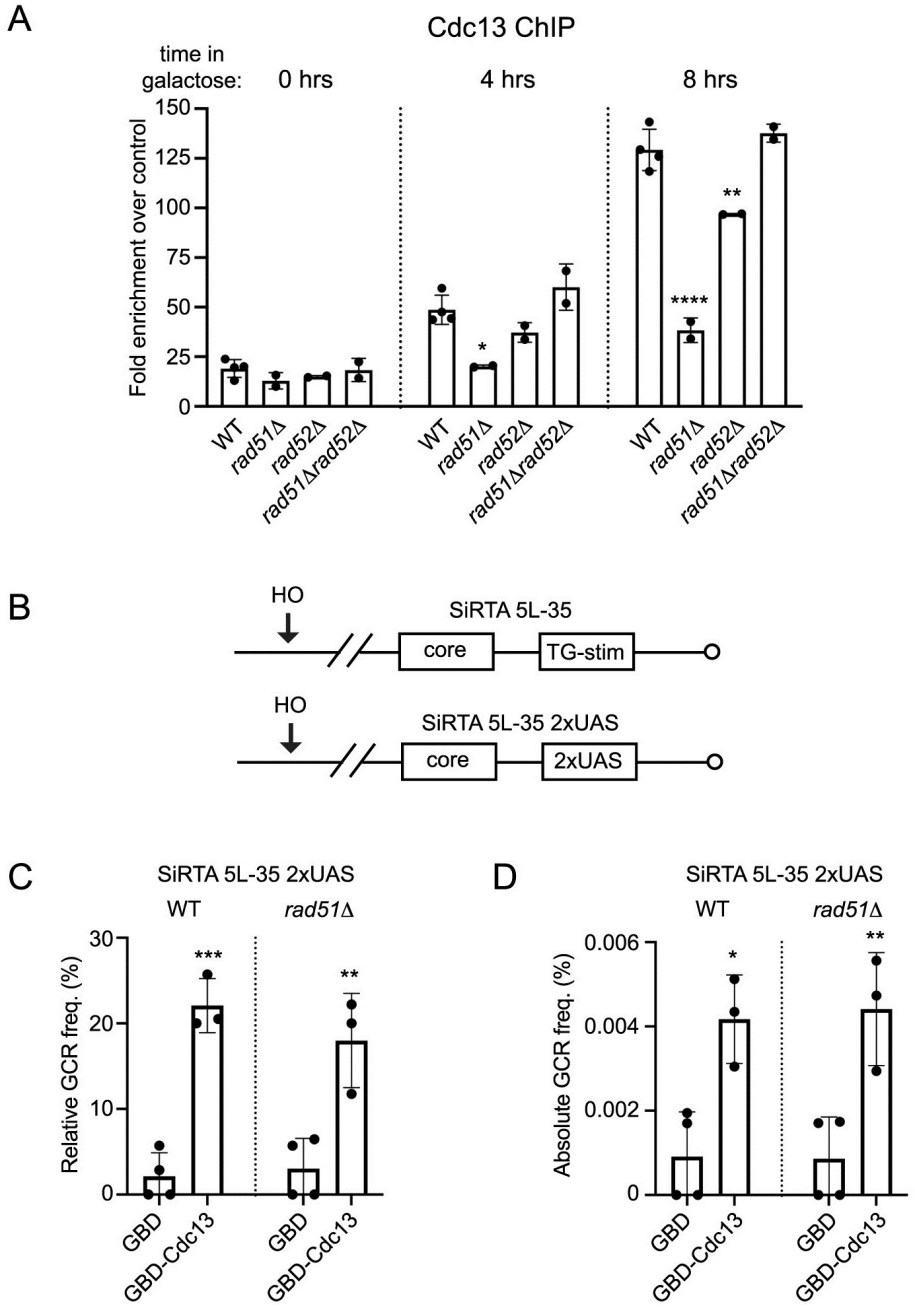
Rad51 promotes the recruitment of Cdc13 to SiRTAs. (A) ChIP analyses of Cdc13 binding at SiRTA 9L-44 in WT, *rad51Δ*, *rad52Δ*, and *rad51Δ rad52Δ* strains are shown for the indicated timepoints following induction of HO cleavage. SiRTA 9L-44 IP signals are normalized to signal at the control *ARO1* locus at the 0 hr timepoint (see Methods). Within a given timepoint, strains that differ significantly from WT by ANOVA with Dunnett’s multiple comparisons test are indicated. (B) Diagram of SiRTA 5L-35 showing the core and TG-stim sequences in an unaltered strain (top). In the experimental strain used in panel C, the TG-stim sequence is replaced with two copies of the Gal4 upstream activating sequence (UAS; bottom). (C) The relative GCR frequency in SiRTA 5L-35 is shown for WT or *rad51Δ* strains containing two copies of the Gal4-UAS sequence integrated in place of the SiRTA-Stim. Cells are transformed with vector expressing GBD only (GBD) or vector expressing full-length Cdc13 fused to the Gal4 DNA binding domain (GBD-Cdc13). Data are averages and standard deviations from at least three independent experiments. Averages indicated by asterisks are statistically different from the corresponding GBD control strains by ANOVA with Tukey’s multiple comparisons test. Average relative GCR frequencies measured in the presence of GBD only or GBD-Cdc13 are not different between the WT and *rad51Δ* backgrounds. (D) The absolute GCR frequency in SiRTA 5L-35 is shown in the indicated strains from the same experiments shown in panel C. Values are averages from at least 3 independent experiments with standard deviation. Averages indicated by asterisks are statistically different from the corresponding GBD control strains by ANOVA with Tukey’s multiple comparisons test. Average absolute GCR frequencies measured in the presence of GBD only or GBD-Cdc13 are not different between the WT and *rad51Δ* backgrounds. (*p<0.05; **p <0.01; ***p<0.001; ****p<0.0001).

To explore this possibility, we monitored recruitment of Cdc13 to the SiRTA 9L-44 locus by chromatin immunoprecipitation (ChIP). We found that while Cdc13 was efficiently recruited in WT cells, recruitment was decreased 2-fold at the 4 hr (p<0.05) and 3-fold at the 8 hr (p<0.0001) timepoints following HO induction in the absence of Rad51 (Fig 6A). Recruitment of Cdc13 was not significantly affected in the absence of Rad52 at the 4 hr timepoint (p = 0.17) and was modestly reduced compared to WT cells at the 8 hr timepoint (p<0.01) (Fig 6A). As expected from our genetic results, deletion of *RAD52* completely suppressed the phenotype of the *rad51Δ* strain, with the double mutant showing association of Cdc13 at levels indistinguishable from WT at both time points (Fig 6A).

If reduced recruitment of Cdc13 observed by ChIP in the *rad51Δ* strain accounts for reduced *de novo* telomere addition at SiRTA, then artificial recruitment of Cdc13 to SiRTA should restore telomere addition in the absence of Rad51. To test this prediction, we utilized a system in which the Stim sequence at SiRTA 5L-35 is replaced with two copies of the Gal4 upstream activating sequence (SiRTA 5L-35 2xUAS; Fig 6B) [20,24]. We previously showed that cells containing SiRTA 5L-35 2xUAS undergo low levels of GCR formation within SiRTA 5L-35, consistent with a requirement for the Stim sequence to achieve high levels of *de novo* telomere addition. In the SiRTA 5L-35 2xUAS background, telomere addition can be stimulated by expression of the Gal4 DNA binding domain (GBD) fused to Cdc13 (GBD-Cdc13), while expression of GBD alone or a fusion of GBD with the telomere-binding protein Rap1 has no effect [20]. Furthermore, the ability of Cdc13-GBD to stimulate telomere addition requires the 2xUAS sequence [20]. To test the effect of forced Cdc13 recruitment in the presence or absence of *RAD51*, SiRTA 5L-35 2xUAS cells (*RAD51* or *rad51Δ*) were transformed with a plasmid expressing either GBD alone or GBD-Cdc13. Consistent with our previous results, expression of GBD alone in the *RAD51* strain supported a very low frequency of GCR formation within SiRTA 5L-35 2xUAS, while GBD-Cdc13 stimulated GCR formation by 5- to 10-fold as measured by either the relative (Fig 6C) or absolute GCR frequency (Fig 6D). When the same experiment was done in cells lacking *RAD51*, two effects were observed. First, in SiRTA 5L-35 2xUAS cells expressing GBD alone, deletion of *RAD51* did not further decrease the frequency of GCR events at the SiRTA, suggesting that Rad51 is only required in the presence of a functional Stim sequence. Second, expression of the GBD-Cdc13 fusion protein rescued GCR formation in the *rad51Δ* strain to the same extent observed in the *RAD51* strain (Fig 6C and 6D). Southern blot analysis of events mapping to SiRTA 5L-35 2xUAS confirmed that 95–100% of GCR events in all four strains involved *de novo* telomere addition (S1 Table). These results are consistent with a model in which Rad51 contributes (perhaps indirectly) to the binding of Cdc13 to SiRTA sequences following HO-induced DSBs.

### Rad52-RPA interaction contributes to suppression of *de novo* telomere addition at SiRTAs

**Fig 7:**
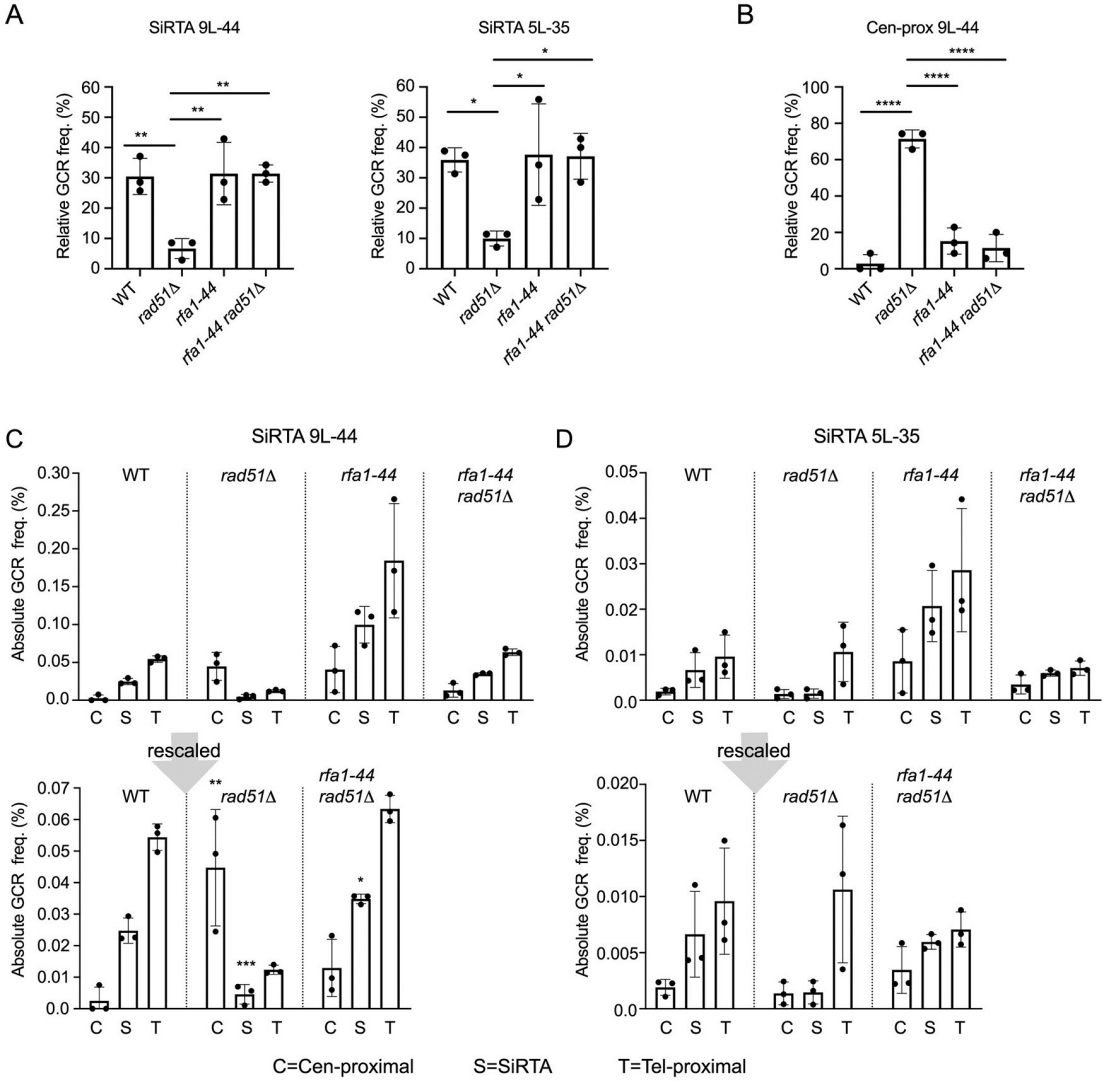
Disruption of the Rad52-Rfa1 interaction suppresses the *de novo* telomere addition defect of *rad51Δ*. (A) The relative GCR frequency in SiRTA 9L-44 and 5L-35 is shown for the indicated strains. (B) The relative GCR frequency in the region centromere-proximal to SiRTA 9L-44 is shown for the same experiments presented in panel A (left graph). Averages and standard deviations are from three independent experiments. Averages indicated by asterisks are statistically different by ANOVA with Tukey’s multiple comparisons test. (C, D) Because the GCR frequency of the *rfa1-44* strain is significantly higher than WT on both chromosomes 5 and 9, the absolute GCR frequency in each region of chromosome 9 (C) or 5 (D) is shown from the same experiments as panel A. Values are averages from three independent experiments with standard deviation. Results for the WT, *rad51Δ*, and *rad51Δ rfa1-44* strains are shown on a different scale underneath. Statistical significance is only shown on the rescaled graph. For the centromere-proximal and SiRTA regions only, averages were compared to the WT sample in that same region by ANOVA with Dunnett’s multiple comparisons test (*p <0.05; **p<0.01; ***p<0.001; ****p<0.0001).

Because the interaction of Rad52 with RPA-bound single-stranded DNA persists in the absence of Rad51 [47], we speculated that the interaction between Rad52 and RPA might be important for the ability of Rad52 to inhibit *de novo* telomere addition. To address this possibility, we tested the effect of a mutation in *RFA1*, the gene encoding the largest subunit of the RPA complex. We chose an allele (*rfa1-44*) that is defective in DSB repair and HO-induced gene conversion and is sensitive to both X-ray and UV irradiation, but does not appear to affect DNA replication since cell growth is relatively unaffected in the absence of DNA damage [48]. The effects of this allele are epistatic with *rad52Δ* and suppressed by overexpression of Rad52, consistent with the mutation disrupting interaction between Rad52 and Rfa1 [49]. A *RAD51* strain carrying the *rfa1-44* mutation at the endogenous *RFA1* locus showed no change in the relative GCR frequency at SiRTAs 9L-44 or 5L-35 compared to WT (Fig 7A). Remarkably, in the absence of *RAD51*, the *rfa1-44* mutation restores the relative frequency of GCR formation at both SiRTAs 9L-44 and 5L-35 (Fig 7A) in a manner equivalent to the complete knockout of *RAD52* (compare with Fig 1B). Furthermore, the *rfa1-44 rad51Δ* strain no longer shows the striking increase in relative GCR frequency in the region centromere-proximal to SiRTA 9L-44 seen upon deletion of *RAD51* alone (Fig 7B). We note that the *rfa1-44* strain incurs GCR events at an overall frequency approximately ~6-7-fold higher than the WT strain, with 20 of 27 events analyzed by Southern blot showing telomere addition (76%). A similar increase in GCR frequency has been reported for other *rfa1* alleles [50]. However, the overall GCR frequency in the *rad51Δ rfa1-44* strain is only slightly higher than the WT range and 24 of 29 GCR events at SiRTA 9L-44 (83%) involve telomere addition. As a consequence, the same patterns described above are still observed when the data are presented as absolute GCR frequencies (Fig 7C and 7D). These results show that the interaction between Rad52 and RPA must be retained for the inhibitory effect of Rad52 on *de novo* telomere addition.

## Discussion

In work described here, we find that Rad51 stimulates *de novo* telomere addition at SiRTAs, endogenous TG-rich sequences previously shown to support unusually high levels of telomere addition [20]. It was previously speculated that formation of the Rad51 nucleoprotein filament on resected 3’ ends may facilitate recruitment of telomerase to DSBs [26], but our data are inconsistent with this model. Surprisingly, we find that Rad52 is not required for *de novo* telomere addition at SiRTAs (Fig 1B and S1 Fig). Indeed, Rad51 nucleoprotein filament formation is dispensable for telomere addition since a variant of Rad51 that cannot bind single-stranded DNA retains normal levels of *de novo* telomere addition (Fig 4A). Furthermore, *de novo* telomere addition is restored by the simultaneous deletion of *RAD51* and *RAD52* (Fig 1B and S1 Fig). This epistatic relationship suggests that Rad52 suppresses *de novo* telomere addition at SiRTAs in a manner that is normally counteracted by Rad51. Our results further show that interaction between Rad51 and Rad52 is required for this regulatory function. Variants of Rad51 that retain DNA binding but are defective for interaction with Rad52 fail to sustain *de novo* telomere addition, as does a mutant version of Rad52 that cannot interact with Rad51 (Fig 4C and 4E). This effect is specific to Rad51 and Rad52, since loss of Rad59, a protein that shares homology with the N-terminal domain of Rad52 but lacks the C-terminal Rad51-interacting domain, neither affects *de novo* telomere addition nor suppresses the effect of deleting *RAD51* (panel A in S2 Fig and Fig 3B). Likewise, mutations in other *RAD52*-epistasis group genes (*RAD54* and *RAD57*) do not consistently reduce telomere addition at SiRTAs (panel A in S2 Fig).

The Chartrand group has reported that that the intranuclear trafficking of the telomerase (TLC1) RNA is modulated in G2/M phase in response to DNA damage [46]. While TLC1 RNA is predominantly nucleolar in G2/M, nucleoplasmic localization increases moderately in response to DNA damage [46]. Rad52 enforces nucleolar localization, which may sequester telomerase from double-strand breaks. In contrast to cells lacking Rad52 function, which display increased nucleoplasmic localization of TLC1 after DNA damage, cells lacking *RAD51* show little relocalization of TLC1 RNA to the nucleoplasm [46] and association of Cdc13 with DNA damage-induced foci is reduced [26,46]. However, while we observe that *rad52Δ* is epistatic to *rad51Δ* for *de novo* telomere addition (Fig 1B), the TLC1 RNA localization phenotype of the *rad51Δ* strain is epistatic to that observed upon loss of *RAD52* [46]. This difference suggests that the effects we see on *de novo* telomere addition at SiRTAs in the absence of *RAD51* and/or *RAD52* cannot be explained by changes in the intranuclear localization of telomerase.

Our data suggest that telomere addition is reduced at SiRTAs in the absence of Rad51 function as a consequence of reduced Cdc13 association with the double-strand break. We find by ChIP that the association of Cdc13 with sequences near the SiRTA on chromosome 9 increases after induction of a double-strand break in WT cells and that binding, while reduced upon deletion of *RAD51*, is rescued by the deletion of *RAD52* (Fig 6A). Importantly, recruitment of the Cdc13-GBD fusion protein completely suppresses the telomere addition defect of the *rad51Δ* strain (Fig 6C), supporting the conclusion that defects in Cdc13 association underlie the reduction in *de novo* telomere addition observed in the absence of Rad51.

How does Rad52 interfere with telomere addition in the absence of Rad51? Based on several *in vitro* observations, we propose the following explanation for our results. Single-molecule experiments reported by the Greene and Sung laboratories show that the association of Rad52 with RPA-coated single-stranded DNA alters the binding properties of RPA [51]. When bound to ssDNA, the RPA complex undergoes transient “micro-dissociation” events that facilitate exchange with other single-stranded DNA binding proteins [52,53]. Remarkably, addition of Rad52 stabilizes the association of RPA with the DNA, rendering RPA resistant to displacement by other proteins in a manner that appears to require direct protein-protein contact between RPA and Rad52 [51]. As expected, addition of Rad51 triggers the Rad52-mediated replacement of RPA with Rad51, although some RPA and Rad52 remain associated with the Rad51 nucleoprotein filaments [51]. Perhaps, when Rad52 interacts with RPA in the absence of Rad51, Cdc13 cannot easily displace RPA. Recruitment of Cdc13 to the SiRTA through binding to the Gal4 UAS (which must happen while the DNA remains double-stranded) would be expected to overcome this deficit, as we observe. We also find that the rfa1-44 variant of RPA prevents Rad52 from inhibiting *de novo* telomere addition in the absence of *RAD51* (Fig 7), consistent with data showing that this mutation reduces the interaction between Rfa1 and Rad52 [48,49].

The single-molecule experiments do not directly address the requirement for the Rad51-Rad52 interaction, nor whether Rad51 protein that lacks the ability to bind DNA can still alter the interaction between Rad52 and RPA. In this regard, Sugiyama and Kantake report that RPA-coated ssDNA is aggregated by the addition of Rad52 in a manner that requires the interaction between Rad52 and Rfa1 [54]. Although it is not entirely clear what these interactions represent, Rad51 leads to the dissolution of the aggregate, even when Rad51 is pre-bound to dsDNA and does not replace RPA on the ssDNA. These results may provide an explanation for the ability of a Rad51 variant that itself cannot bind ssDNA to disrupt the inhibitory effect of Rad52 on *de novo* telomere addition, perhaps by altering the interaction of Rad52 with RPA and/or the ability of Rad52 to self-associate.

Although we were initially interested in understanding the influence of HR-associated proteins on *de novo* telomere addition, we observed a striking increase in the fraction of repair events occurring internal to the SiRTA on chromosome 9 upon deletion of *RAD51*, suggesting that these events are normally inhibited by Rad51 function (Fig 2A). This change does not result directly from reduced repair at the SiRTA because complete deletion of the SiRTA sequence is not associated with increased repair in the centromere-proximal region (Fig 2B). Furthermore, the effects at the SiRTA and the internal region are genetically separable—deletion of *RAD59* in the *rad51Δ* background suppresses the events in the centromere-proximal region, but does not restore telomere addition at the SiRTA (Fig 3B).

To gain insight into the nature of these events, we used nanopore technology to sequence the entire genomes of twelve independent survivors of HO cleavage (in a *rad51Δ* background). Three of the events were non-reciprocal translocations that in each case were mediated by small (<25 bp) regions of microhomology (Fig 3A). Since the intact chromosome was detected in each case and all events occurred within 75 kb of a telomere, these events likely arose through BIR, a repair event in which single-stranded DNA produced on the proximal side of a DSB invades a homologous duplex with subsequent extension of the 3’ end in a conservative manner using the invaded strand as template [13]. Consistent with this idea, we find that the increase in centromere-proximal events in the *rad51Δ* background depends strongly on *RAD52*, *RAD59*, and *POL32* (Figs 2A and 3B–3D), all genes shown previously to contribute to BIR [34,37].

The most common event observed was an internal deletion, with eight of twelve strains showing the identical event, again between a short (19 bp) region of imperfect micro-homology. Having identified this deletion, we analyzed a large number of centromere-proximal events accumulated from the experiments described here and found that the frequency of this particular deletion increases dramatically in the absence of *RAD51*. While it is formally possible that the large internal deletions on chromosome 9 are mediated by BIR (with an intrachromosomal invasion event), we favor the idea that these events occur through a variation of microhomology-mediated recombination (MHMR) characterized in detail by Villarreal *et al*. on model substrates [36]. Such events resemble SSA, but are distinguished by the use of very short (15–18 bp) regions of microhomology and strong dependence on *POL32*. Congruent with our observations, *RAD52* and *RAD59* were shown to stimulate MHMR when microhomologies are 15–18 bp in length, while *RAD51* represses these events [36]. Remarkably, the 39 kb deletion is the result of repair between two micro-homologous sequences that are located 12 kb proximal and 27 kb distal to the site of HO cleavage, demonstrating that such events can occur even after extensive 5’ end resection. Our observation that interaction between Rad51 and Rad52 is required to fully repress these SSA-like events is consistent with *in vitro* experiments demonstrating that interaction with Rad51 suppresses the DNA-annealing activity of Rad52 [36].

It is intriguing that we do not see a significant increase in centromere-proximal events on chromosome 5. This difference may reflect the relative sizes of the two regions (the distance between the SiRTA and the first essential gene is nearly two times greater on chromosome 9; Fig 1A) or the fortuitous existence of microhomologies. We note, however, that all of the events we sequenced on chromosome 9 have proximal breakpoints within 5 kb of each other, even though the centromere-proximal region is nearly 17 kb. This observation is reminiscent of a report from the Haber lab of an enhancer on chromosome 3 that greatly stimulates SSA within a neighboring region [55]. On chromosome 3, the enhancing sequence was mapped to a 200 bp region adjacent to, but not including, an origin of replication [55]. There is no origin reported in the region immediately proximal to the breakpoint junctions and no obvious sequence homology with the enhancer on chromosome 3. Future studies are required to determine if such enhancing activity is present on chromosome 9.

## Methods

### Yeast strains and plasmids

All strains used in this study are listed in S2 Table and are derivatives of YKF1308 and YKF1310 [20,29]. Unless otherwise indicated, strains were grown in yeast extract/peptone/dextrose medium (YEPD) at 30°C. All gene deletions were derived by one-step gene replacement using a selectable marker and verified by PCR.

Mutations in the *RAD51* gene (*rad51-Y388H*, *rad51-G393D* and *rad51-L99P*) were introduced by replacing a portion of *RAD51* with *URA3* in strain YKF1508 by one-step gene replacement to create strain YKF1508+URA3. Sequences containing desired mutations were generated by a two-step PCR reaction and PCR products were transformed into YKF1508+URA3. Cells were allowed to recover on rich media overnight and then replica-plated onto medium containing 5-FOA. Candidates were confirmed by sequencing.

*RAD52*, *RFA1*, *rad51-K191A* and *rad51-K191R* alleles were constructed utilizing the CRISPR-Cas9 cleavage system. gRNAs were designed and cloned into a *BpI*I-digested plasmid bRA90 constitutively expressing Cas9 [56] (a gift from Dr. James Haber). Plasmids were verified by PCR and co-transformed into yeast strains with PCR-derived linear DNAs containing the desired mutations. Candidates surviving selection for the bRA90 plasmid were verified by sequencing and screened for loss of bRA90 prior to use.

### Inducible HO cleavage assay

Yeast cells were grown overnight in synthetic complete media lacking uracil (SC-Ura) containing 2% raffinose to OD_600_ of ~0.6 to 0.8. 10–30 μl aliquots of culture were plated on yeast extract/peptone medium containing 2% galactose (YEPG) and a dilution was plated on rich medium containing 2% glucose (YEPD) to determine total viable cell count. Unless otherwise noted, plates were incubated at 30°C for 3 days. Surviving colonies were counted and at least 100 galactose-resistant (Gal^R^) colonies were patched to plates containing 5-FOA to isolate GCR events (Gal^R^ 5-FOA^R^ colonies). Additional Gal^R^5-FOA^R^ colonies were obtained by replica plating where necessary. For each experiment, at least 30 Gal^R^ 5-FOA^R^ colonies were analyzed to determine the approximate location and/or type of GCR event; averages and standard deviations were derived from a minimum of three independent experiments except *yku80Δ* where two experiments were analyzed. The location and nature of GCR events were determined by PCR and Southern blotting as previously described [20] and as described below. The relative frequency of GCR formation in a particular region is determined by dividing the number of events observed in that region by the total number of Gal^R^ 5-FOA^R^ events. The absolute frequency at which GCR formation occurs in a particular region is derived by multiplying the frequency at which Gal^R^ colonies are produced (surviving colonies on galactose plates/surviving colonies on glucose plates, adjusted for dilution) by the fraction of Gal^R^ colonies that survive on media containing 5-FOA (Gal^R^ 5-FOA^R^) and by the fraction of Gal^R^ 5-FOA^R^ colonies that map to that particular region (the relative frequency of GCR formation) [20]. The overall GCR frequency is achieved by multiplying the frequency of Gal^R^ colonies by the fraction of Gal^R^ colonies that survive on media containing 5-FOA (Gal^R^ 5-FOA^R^). See Fig 1C.

For each experiment, the overall GCR frequency for each strain was compared with the frequencies obtained in multiple measurements of the *RAD51* and *rad51Δ* strains following cleavage of the appropriate chromosome. When values are not statistically different by ANOVA with Tukey’s multiple comparisons test, then relative GCR frequencies of events in the centromere-proximal and SiRTA regions are presented. In cases where significant differences are observed, absolute GCR frequencies are additionally presented. Overall GCR frequencies are given in S2 Data.

### Southern blotting

Total genomic DNA was extracted by glass beads lysis [57]. Extracted DNA was digested overnight with *Nsi*I (New England Biolabs) or *Alw*NI (NEB) to analyze events occurring at SiRTA 9L-44 or 5L-35 respectively or *Xho*I (NEB) to analyze endogenous telomeres. Digested fragments were separated on a 0.7% agarose gel and subsequently denatured. Denatured fragments were transferred to nylon membrane (Amersham Hybond N+; GE Healthcare) overnight and prehybridized for several hours. Pre-hybridized membranes were probed overnight with [^32^P]dCTP-labelled, random-primed DNA derived from PCR amplification of the SiRTA 9L-44 locus or telomeric DNA to detect SiRTA 9L-44 or endogenous telomeres respectively. In some cases, membranes were stripped by boiling in 0.1% SDS solution and reprobed with telomeric DNA to detect endogenous telomeres. Following hybridization, membranes were washed and exposed to Phosphor screens (Molecular Dynamics) and screens were scanned with Typhoon TRIO variable mode imager (GE Healthcare). Primers used to generate probes for Southern blot analysis of GCRs are listed in S3 Table.

### Identification of chromosome breakpoints

Genomic DNA was isolated using the MasterpureTM Yeast DNA purification kit (Lucigen) and sequenced using MinION technology from Oxford Nanopore at VANTAGE (Vanderbilt Technologies for Advanced Genomics). Libraries from twelve samples were generated for MinION sequencing using the Rapid Barcoding Kit (RBK-004) per manufacturer’s instructions (Oxford Nanopore). Sequencing was performed on a total of 3 flow cells (type R9.4.1). Sequences were demultiplexed using porechop v0.2.4 (https://github.com/rrwick/Porechop) with default settings.

Sequence reads were analyzed with tools available at UseGalaxy.org. Briefly, each dataset was converted to a BLAST database using NCBI BLAST+ makeblastdb [58,59], which allowed a list of reads matching chromosome 9 sequences centromere-proximal to the SiRTA to be generated. Iterative BLAST searches with the tool in the Saccharomyces Genome Database (SGD; https://www.yeastgenome.org) were used to pinpoint the site of the rearrangement and/or to identify sequence reads containing intact chromosomes. Breakpoints were verified by PCR. Primer sequences and additional information about each of the 12 clones can be found in supplementary data (S1 Data). All sequence reads obtained have been submitted to the NCBI Sequence Read Archive (SRA) under BioProject accession number PRJNA557764.

### Chromatin immunoprecipitation

Yeast cells were grown in yeast extract/peptone with 2% raffinose to OD_600_ of 0.5 to 0.6. DSB induction was achieved by adding galactose to a final concentration of 2%. At the indicated timepoints, 50 ml of cells were removed and fixed in formaldehyde to final concentration of 1% at room temperature for 30 min. At later timepoints, cells were diluted with media to maintain the initial OD_600_ reading. Quenching was done by adding glycine to a final concentration of 125 mM for 5 min at room temperature. Cells were washed twice with ice-cold HBS (50 mM HEPES at pH 7.6, 140 mM NaCl), frozen in dry ice and stored at -80°C. Cell pellets were resuspended in 400 μl ChIP lysis buffer high salt (Santa Cruz Biotechnologies; sc-45001) containing protease inhibitor cocktail (Roche; 1 tablet/5 ml ChIP lysis buffer) and lysed with an equal volume of glass beads at 4°C by vortexing at maximum speed for 40 min. Cell lysates were sonicated on the Covaris LE220 series with settings 450 peak power, 30% Duty factor, 200 cycles per burst for 15 min in AFA crimp-cap 130 μl tubes (Covaris) to yield an average fragment size of 0.1 to 0.5 kb. Sonicated lysates were clarified by centrifugation twice (13, 000 rpm for 5 min, then 13,000 rpm for 15 min), supernatant was transferred to a new tube after each centrifugation. A portion of the pre-IP extract was set aside as input sample. The remaining extracts were incubated with 6 μg of anti-myc antibody (Roche; 11667149001) at 4°C overnight. Protein-G magnetic beads (Life Technologies) were added to each sample and incubation continued for 4–6 hours. Beads were washed 2 times with ChIP lysis buffer (Santa Cruz Biotechnologies; sc-45000), 3 times with ChIP lysis buffer high salt (Santa Cruz Biotechnologies; sc-45001) and ChIP wash buffer (Santa Cruz Biotechnologies; sc-45002) at 4°C for 5 min each. Immunoprecipitated chromatin was eluted in ChIP elution buffer (50 mM Tris-Hcl pH 7.5, 10 mM EDTA, 1% SDS, 200 mM NaCl) at 65°C for 30 min. Reversal of crosslinks was carried out at 65°C for 14 hr. All samples (pre-IP and IP eluates) were treated with RNase for 1 hr at 37°C and proteinase K for 2hrs at 55°C. DNA was purified by phenol:chloroform:isoamy alcohol (24:25:1) extraction, followed by ethanol precipitation. Purified DNA was resuspended in buffer EB (Qiagen) and used in qPCR reactions containing 1X SsoAdvanced universal SYBR green supermix (Bio-Rad) and 500 nM of each primer. DNA in each sample was quantified by comparison to a standard curve generated from a dilution of sonicated yeast genomic DNA. qPCR reactions were carried out in a C1000 Thermal Cycler with CFX96 Real-time System (Bio-Rad) and data were analyzed using CFX Manger software (Bio-Rad). Primer sequences are provided in supplementary information (S3 Table). The amount of IP DNA at the SiRTA 9L-44 locus is divided by the respective time point input DNA from an independent ARO1 locus to correct for the progressive loss of input DNA at the SiRTA locus. The IP SiRTA 9L-44/ARO1 input ratio at each time point is then normalized to the IP ARO1/Input ARO1 signal before HO induction [60,61].

### Western blot

Strains were grown in YPD at 30°C to OD_600_ ~1. Cells were washed once with cold ddH_2_0 and TMG (10 mM Tris-Cl at pH 8, 1 mM MgCl_2_, 10% glycerol, 0.1 mM DTT) with 200 mM NaCl and subsequently lysed with glass beads in 1X IP extraction buffer (Thermofisher Dynabeads Co-immunoprecipitation kit, 14321D) supplemented with 1 mM MgCl_2_, 0.1 mM DTT and 200 mM NaCl. One complete, mini protease inhibitor tablet (Roche, 4693159001) was added to each 10 ml of lysis buffer. Extracts were normalized to 25 mg/ml and incubated with 4 μg of anti-Rad51 antibody (Abcam 63798) overnight at 4°C. 80 μl of protein-G dynabeads (Thermoscientific) was added and incubation continued for another 5 hours. Beads were washed three times with IP extraction buffer and once with 1X LWB (Thermofisher Dynabeads co-immunoprecipitation kit) for 5 min. Immunoprecipitation beads were mixed with 2X Laemmli loading buffer (Bio-Rad). 25 μl of immunoprecipitated beads (1/2 of total) was heated to 100°C for 5 min and supernatant separated on 7.5% Bio-Rad TGX stain-free gels. Proteins were transferred to membrane (Bio-Rad trans-blot mini PVDF transfer kit) using the Bio-Rad trans-blot turbo transfer system. The membrane was blocked with intercept (TBS) blocking buffer (LI-COR) for 1 hour at room temperature. The membrane was cut slightly below the 75 kDa mark and the upper portion was incubated with anti-myc (1:1000 Roche, 1166714149001) while the lower portion was incubated with anti-Rad51 antibody (1:2000, Abcam 63798) in blocking buffer overnight at 4°C. Membrane was washed four times with 1X TBS-T followed by secondary antibody incubation (LI-COR anti-mouse or anti-rabbit 680LT) used at a 1:10,000 dilution for 1 hour at room temperature. Membrane was washed four times with 1X TBS-T and imaged on the Bio-Rad Chemidoc imaging system.

### Adaptation

Adaptation experiments were performed essentially as described in [39]. Cells were grown overnight in non-inducing medium [yeast extract-peptone (YP) with 2% raffinose] at 30°C and spread on a YP 2% galactose plate. Single unbudded cells were micromanipulated in a grid, plates were incubated at 30°C, and the number of cells per microcolony was counted at 24 hours after plating. A small number of cells that never divided were not included in the analysis.

## Supporting information

S1 Fig Loss of Rad51 alters the distribution of repair events in and centromere-proximal to the SiRTA. (A) The relative GCR frequency in each region on chromosome 9 (left) or chromosome 5 (right) is shown in the indicated strains. C, S, and T indicate centromere-proximal, SiRTA, and telomere-proximal events, respectively. Data are from the same experiments shown in Figs 1B and 2A. Values are averages of at least three independent experiments with standard deviation. For the centromere-proximal and SiRTA regions only, averages were compared to the WT sample in that same region by ANOVA with Dunnett’s multiple comparisons test. (B) The absolute GCR frequency (see Fig 1C for calculation) in each region on chromosome 9 (left) or 5 (right) is shown in the indicated strains from the same experiments as panel A. Values are averages from three independent experiments with standard deviation. For the centromere-proximal and SiRTA regions only, averages were compared to the WT sample in that same region by ANOVA with Dunnett’s multiple comparisons test (*p<0.05; **p <0.01; ***p<0.001; ****p<0.0001).
(PDF)

S2 Fig The distribution of GCR events is unaffected by loss of Rad54, Rad55, Rad57 and Rad59. (A) The relative GCR frequency in SiRTA 9L-44 and 5L-35 is shown for the indicated strains. (B) The relative GCR frequency in the region centromere-proximal to SiRTA 9L-44 and 5L-35 is shown for the same experiments in panel A. Averages of at least three independent experiments are shown with standard deviation. Values statistically different from WT by ANOVA with Dunnett’s multiple comparisons test are indicated by asterisks (*p <0.05). Overall GCR frequencies of the strains analyzed in this figure did not differ significantly from those measured in the *RAD51* and *rad51Δ* strains within the same chromosome region.
(PDF)

S3 Fig Endogenous telomere lengths are not altered by deletion of *RAD51* and/or *RAD52*. Southern blot analysis endogenous telomeres in WT, *rad51Δ*, *rad52Δ*, and *rad51Δ rad52Δ* strains. 9L-44 and 5L-35 indicate the YKF1752 and YKF1342 strain backgrounds, respectively (S2 Table). The first and last lanes contain molecular weight marker as indicated.
(PDF)

S4 Fig Southern blot analysis of GCR events occurring within SiRTA 9L-44. (A) Diagram of the region of chromosome 9 surrounding SiRTA 9L-44 in a WT strain (top) or a strain that has undergone *de novo* telomere addition at SiRTA 9L-44 (bottom). Sites of cleavage by *Nsi*I and the probes utilized in panel B (SiRTA probe) and C (telomere probe) are shown. (B) A Southern blot conducted on 17 independent GCR events that mapped to SiRTA 9L-44 by PCR (lanes 2–18) was probed with a PCR product located immediately centromere-proximal to SiRTA 9L-44 (see panel A). Lane 19 contains DNA isolated from a WT strain before HO cleavage. Lanes 1 and 20 contain molecular weight marker as indicated. Telomere addition events are indicated with asterisks (*). (C) The same blot shown in B was stripped and reprobed with a short fragment of yeast telomeric DNA. Although multiple bands are detected, the same fragments indicated in panel B are evident (*).
(PDF)

S5 Fig Association of Rad51 with Rad52 assayed by immunoprecipitation. Whole cell protein extracts were generated from strains expressing the indicated *RAD51* alleles. Strains contained Myc-tagged *RAD52* with the exception of the strain in lane 2. Left panel: Whole cell extracts were probed with anti-Rad51 (top) or anti-Myc (middle) antibodies. Prior to blotting, total protein load was assessed (bottom). Right panel: The same extracts were immunoprecipitated using the anti-Rad51 antibody and probed for Rad51 (top) or Myc (bottom). Sizes of molecular weight markers are indicated (kilodaltons). The L99P strain contains fewer Myc epitopes than the other strains as determined by PCR of the genomic DNA, resulting in slightly faster migration of the Rad52-Myc protein.
(PDF)

S1 Data Summary of nanopore sequencing data obtained for 12 GCR events in the *rad51Δ* background. (PDF)

S2 Data Data file corresponding to all graphs of this manuscript. (XLSX)

S1 Table Frequency of telomere addition for GCR events occurring at SiRTAs. (PDF)

S2 Table List of strains. (PDF)

S3 Table List of primers for chromatin immunoprecipitation. (PDF)

## References

[1] Wellinger RJ, Zakian VA. Everything you ever wanted to know about *Saccharomyces cerevisiae* telomeres: beginning to end. Genetics. 2012;191(4):1073–105. 10.1534/genetics.111.137851 22879408 PMC3415994

[2] Osterhage JL, Friedman KL. Chromosome end maintenance by telomerase. J Biol Chem. 2009;284(24):16061–5. 10.1074/jbc.R900011200 19286666 PMC2713563

[3] Mehta A, Haber JE. Sources of DNA double-strand breaks and models of recombinational DNA repair. Cold Spring Harb Perspect Biol. 2014;6(9):a016428 10.1101/cshperspect.a016428 25104768 PMC4142968

[4] Aylon Y, Kupiec M. DSB repair: the yeast paradigm. DNA Repair (Amst). 2004;3(8–9):797–815. 10.1016/j.dnarep.2004.04.013 15279765

[5] Brill SJ, Stillman B. Replication factor-A from *Saccharomyces cerevisiae* is encoded by three essential genes coordinately expressed at S phase. Genes Dev. 1991;5(9):1589–600. 10.1101/gad.5.9.1589 1885001

[6] Wang X, Haber JE. Role of *Saccharomyces* single-stranded DNA-binding protein RPA in the strand invasion step of double-strand break repair. PLoS Biol. 2004;2(1):E21 10.1371/journal.pbio.0020021 14737196 PMC314472

[7] Sugiyama T, Kowalczykowski SC. Rad52 protein associates with replication protein A (RPA)-single-stranded DNA to accelerate Rad51-mediated displacement of RPA and presynaptic complex formation. J Biol Chem. 2002;277(35):31663–72. 10.1074/jbc.M203494200 12077133

[8] New JH, Sugiyama T, Zaitseva E, Kowalczykowski SC. Rad52 protein stimulates DNA strand exchange by Rad51 and replication protein A. Nature. 1998;391(6665):407–10. 10.1038/34950 9450760

[9] Sung P, Krejci L, Van Komen S, Sehorn MG. Rad51 recombinase and recombination mediators. J Biol Chem. 2003;278(44):42729–32. 10.1074/jbc.R300027200 12912992

[10] Mortensen UH, Bendixen C, Sunjevaric I, Rothstein R. DNA strand annealing is promoted by the yeast Rad52 protein. Proc Natl Acad Sci U S A. 1996;93(20):10729–34. 10.1073/pnas.93.20.10729 8855248 PMC38223

[11] Sugiyama T, New JH, Kowalczykowski SC. DNA annealing by RAD52 protein is stimulated by specific interaction with the complex of replication protein A and single-stranded DNA. Proc Natl Acad Sci U S A. 1998;95(11):6049–54. 10.1073/pnas.95.11.6049 9600915 PMC27583

[12] McVey M, Lee SE. MMEJ repair of double-strand breaks (director’s cut): deleted sequences and alternative endings. Trends Genet. 2008;24(11):529–38. 10.1016/j.tig.2008.08.007 18809224 PMC5303623

[13] Kramara J, Osia B, Malkova A. Break-Induced Replication: The where, the why, and the how. Trends Genet. 2018;34(7):518–31. 10.1016/j.tig.2018.04.002 29735283 PMC6469874

[14] Verma P, Greenberg RA. Noncanonical views of homology-directed DNA repair. Genes Dev. 2016;30(10):1138–54. 10.1101/gad.280545.116 27222516 PMC4888836

[15] Putnam CD, Pennaneach V, Kolodner RD. Chromosome healing through terminal deletions generated by *de novo* telomere additions in *Saccharomyces cerevisiae*. Proc Natl Acad Sci U S A. 2004;101(36):13262–7. 10.1073/pnas.0405443101 15328403 PMC516557

[16] Putnam CD, Kolodner RD. Pathways and Mechanisms that Prevent Genome Instability in *Saccharomyces cerevisiae*. Genetics. 2017;206(3):1187–225. 10.1534/genetics.112.145805 28684602 PMC5500125

[17] Myung K, Chen C, Kolodner RD. Multiple pathways cooperate in the suppression of genome instability in *Saccharomyces cerevisiae*. Nature. 2001;411(6841):1073–6. 10.1038/35082608 11429610

[18] Zhang W, Durocher D. *De novo* telomere formation is suppressed by the Mec1-dependent inhibition of Cdc13 accumulation at DNA breaks. Genes Dev. 2010;24(5):502–15. 10.1101/gad.1869110 20194442 PMC2827845

[19] Stellwagen AE, Haimberger ZW, Veatch JR, Gottschling DE. Ku interacts with telomerase RNA to promote telomere addition at native and broken chromosome ends. Genes Dev. 2003;17(19):2384–95. 10.1101/gad.1125903 12975323 PMC218076

[20] Obodo UC, Epum EA, Platts MH, Seloff J, Dahlson NA, Velkovsky SM, et al Endogenous hot spots of *de novo* telomere addition in the yeast genome contain proximal enhancers that bind Cdc13. Mol Cell Biol. 2016;36(12):1750–63. 10.1128/MCB.00095-16 27044869 PMC4907100

[21] Singer M, Gottschling D. *TLC1*: template RNA component of *Saccharomyces cerevisiae* telomerase. Science. 1994;266(5184):404–9. 10.1126/science.7545955 7545955

[22] Pennock E, Buckley K, Lundblad V. Cdc13 delivers separate complexes to the telomere for end protection and replication. Cell. 2001;104(3):387–96. 10.1016/s0092-8674(01)00226-4 11239396

[23] Evans SK, Lundblad V. Est1 and Cdc13 as comediators of telomerase access. Science. 1999;286(5437):117–20. 10.1126/science.286.5437.117 10506558

[24] Bianchi A, Negrini S, Shore D. Delivery of yeast telomerase to a DNA break depends on the recruitment functions of Cdc13 and Est1. Mol Cell. 2004;16(1):139–46. 10.1016/j.molcel.2004.09.009 15469829

[25] Nugent CI, Hughes TR, Lue NF, Lundblad V. Cdc13p: a single-strand telomeric DNA-binding protein with a dual role in yeast telomere maintenance. Science. 1996;274(5285):249–52. 10.1126/science.274.5285.249 8824190

[26] Oza P, Jaspersen SL, Miele A, Dekker J, Peterson CL. Mechanisms that regulate localization of a DNA double-strand break to the nuclear periphery. Genes Dev. 2009;23(8):912–27. 10.1101/gad.1782209 19390086 PMC2675867

[27] Makovets S, Blackburn EH. DNA damage signalling prevents deleterious telomere addition at DNA breaks. Nat Cell Biol. 2009;11(11):1383–6. 10.1038/ncb1985 19838171 PMC2806817

[28] Strecker J, Stinus S, Caballero MP, Szilard RK, Chang M, Durocher D. A sharp Pif1-dependent threshold separates DNA double-strand breaks from critically short telomeres. Elife. 2017;6 10.7554/eLife.23783 28826474 PMC5595431

[29] Lydeard JR, Lipkin-Moore Z, Jain S, Eapen V V., Haber JE. Sgs1 and Exo1 redundantly inhibit break-induced replication and *de novo* telomere addition at broken chromosome ends. PLoS Genet. 2010;6(5):25 10.1371/journal.pgen.1000973 20523895 PMC2877739

[30] Teste M-A, François JM, Parrou J-L. Characterization of a new multigene family encoding isomaltases in the yeast *Saccharomyces cerevisiae*, the IMA family. J Biol Chem. 2010;285(35):26815–24. 10.1074/jbc.M110.145946 20562106 PMC2930680

[31] Bai Y, Symington LS. A Rad52 homolog is required for *RAD51*-independent mitotic recombination in *Saccharomyces cerevisiae*. Genes Dev. 1996;10(16):2025–37. 10.1101/gad.10.16.2025 8769646

[32] Sugawara N, Ira G, Haber JE. DNA length dependence of the single-strand annealing pathway and the role of *Saccharomyces cerevisiae RAD59* in double-strand break repair. Mol Cell Biol. 2000;20(14):5300–9. 10.1128/mcb.20.14.5300-5309.2000 10866686 PMC85979

[33] Jablonovich Z, Liefshitz B, Steinlauf R, Kupiec M. Characterization of the role played by the *RAD59* gene of *Saccharomyces cerevisiae* in ectopic recombination. Curr Genet. 1999;36(1–2):13–20. 10.1007/s002940050467 10447590

[34] Signon L, Malkova A, Naylor ML, Klein H, Haber JE. Genetic requirements for *RAD51*- and *RAD54*-independent break-induced replication repair of a chromosomal double-strand break. Mol Cell Biol. 2001;21(6):2048–56. 10.1128/MCB.21.6.2048-2056.2001 11238940 PMC86809

[35] Gerik KJ, Li X, Pautz A, Burgers PMJ. Characterization of the two small subunits of *Saccharomyces cerevisiae* DNA polymerase δ. J Biol Chem. 1998;273(31):19747–55. 10.1074/jbc.273.31.19747 9677405

[36] Villarreal DD, Lee K, Deem A, Shim EY, Malkova A, Lee SE. Microhomology directs diverse DNA break repair pathways and chromosomal translocations. PLoS Genet. 2012;8(11):e1003026 10.1371/journal.pgen.1003026 23144625 PMC3493447

[37] Lydeard JR, Jain S, Yamaguchi M, Haber JE. Break-induced replication and telomerase-independent telomere maintenance require Pol32. Nature. 2007;448(7155):820–3. 10.1038/nature06047 17671506

[38] Sung P, Stratton SA. Yeast Rad51 recombinase mediates polar DNA strand exchange in the absence of ATP hydrolysis. J Biol Chem. 1996;271(45):27983–6. 10.1074/jbc.271.45.27983 8910403

[39] Lee SE, Pellicioli A, Vaze MB, Sugawara N, Malkova A, Foiani M, et al Yeast Rad52 and Rad51 recombination proteins define a second pathway of DNA damage assessment in response to a single double-strand break. Mol Cell Biol. 2003;23(23):8913–23. 10.1128/MCB.23.23.8913-8923.2003 14612428 PMC262690

[40] Fung CW, Fortin GS, Peterson SE, Symington LS. The *rad51-K191R* ATPase-defective mutant Is impaired for presynaptic filament formation. Mol Cell Biol. 2006;26(24):9544–54. 10.1128/MCB.00599-06 17030607 PMC1698519

[41] Krejci L, Damborsky J, Thomsen B, Duno M, Bendixen C. Molecular dissection of interactions between Rad51 and members of the recombination-repair group. Mol Cell Biol. 2001;21(3):966–76. 10.1128/MCB.21.3.966-976.2001 11154282 PMC86686

[42] Seong C, Colavito S, Kwon Y, Sung P, Krejci L. Regulation of Rad51 recombinase presynaptic filament assembly via interactions with the Rad52 mediator and the Srs2 anti-recombinase. J Biol Chem. 2009;284(36):24363–71. 10.1074/jbc.M109.032953 19605344 PMC2782029

[43] Krejci L, Song B, Bussen W, Rothstein R, Mortensen UH, Sung P. Interaction with Rad51 is indispensable for recombination mediator function of Rad52. J Biol Chem. 2002;277(42):40132–41. 10.1074/jbc.M206511200 12171935

[44] Lee SE, Pellicioli A, Malkova A, Foiani M, Haber JE. The *Saccharomyces* recombination protein Tid1p is required for adaptation from G2/M arrest induced by a double-strand break. Curr Biol. 2001;11(13):1053–7. 10.1016/s0960-9822(01)00296-2 11470411

[45] Lee SE, Moore JK, Holmes A, Umezu K, Kolodner RD, Haber JE. *Saccharomyces* Ku70, Mre11/Rad50, and RPA proteins regulate adaptation to G2/M arrest after DNA damage. Cell. 1998;94(3):399–409. 10.1016/s0092-8674(00)81482-8 9708741

[46] Ouenzar F, Lalonde M, Laprade H, Morin G, Gallardo F, Tremblay-Belzile S, et al Cell cycle-dependent spatial segregation of telomerase from sites of DNA damage. J Cell Biol. 2017;216(8):2355–71. 10.1083/jcb.201610071 28637749 PMC5551704

[47] Miyazaki T, Bressan DA, Shinohara M, Haber JE, Shinohara A. *In vivo* assembly and disassembly of Rad51 and Rad52 complexes during double-strand break repair. EMBO J. 2004;23(4):939–49. 10.1038/sj.emboj.7600091 14765116 PMC380999

[48] Firmenich AA, Elias-Arnanz M, Berg P. A novel allele of *Saccharomyces cerevisiae RFA1* that is deficient in recombination and repair and suppressible by *RAD52*. Mol Cell Biol. 1995;15(3):1620–31. 10.1128/mcb.15.3.1620 7862153 PMC230386

[49] Hays SL, Firmenich AA, Massey P, Banerjee R, Berg P. Studies of the interaction between Rad52 protein and the yeast single-stranded DNA binding protein RPA. Mol Cell Biol. 1998;18(7):4400–6. 10.1128/mcb.18.7.4400 9632824 PMC109024

[50] Chen C, Umezu K, Kolodner RD. Chromosomal rearrangements occur in *S*. *cerevisiae rfa1* mutator mutants due to mutagenic lesions processed by double-strand-break repair. Mol Cell. 1998;2(1):9–22. 10.1016/s1097-2765(00)80109-4 9702187

[51] Gibb B, Ye LF, Kwon Y, Niu H, Sung P, Greene EC. Protein dynamics during presynaptic complex assembly on individual ssDNA molecules. Nat Struct Mol Biol. 2014;21(10):893 10.1038/nsmb.2886 25195049 PMC4190069

[52] Gibb B, Ye LF, Gergoudis SC, Kwon Y, Niu H, Sung P, et al Concentration-dependent exchange of replication protein A on single-stranded DNA revealed by single-molecule imaging. PLoS One. 2014;9(2):e87922 10.1371/journal.pone.0087922 24498402 PMC3912175

[53] Deng SK, Gibb B, de Almeida MJ, Greene EC, Symington LS. RPA antagonizes microhomology-mediated repair of DNA double-strand breaks. Nat Struct Mol Biol. 2014;21(4):405–12. 10.1038/nsmb.2786 24608368 PMC3980576

[54] Sugiyama T, Kantake N. Dynamic regulatory interactions of Rad51, Rad52, and Replication Protein-A in recombination intermediates. J Mol Biol. 2009;390(1):45–55. 10.1016/j.jmb.2009.05.009 19445949

[55] Malkova A, Signon L, Schaefer CB, Naylor ML, Theis JF, Newlon CS, et al *RAD51*-independent break-induced replication to repair a broken chromosome depends on a distant enhancer site. Genes Dev. 2001;15(9):1055–60. 10.1101/gad.875901 11331601 PMC312680

[56] Anand R, Memisoglu G, Haber J. Cas9-mediated gene editing in *Saccharomyces cerevisiae*. Protoc Exch. 2017.

[57] Radford A. Methods in yeast genetics—A laboratory course manual. Biochem Educ. 1991;19(2):101–2.

[58] Cock PJA, Chilton JM, Grüning B, Johnson JE, Soranzo N. NCBI BLAST+ integrated into Galaxy. Gigascience. 2015;4(1):39 10.1186/s13742-015-0080-7 26336600 PMC4557756

[59] Camacho C, Coulouris G, Avagyan V, Ma N, Papadopoulos J, Bealer K, et al BLAST+: architecture and applications. BMC Bioinformatics. 2009;10(1):421 10.1186/1471-2105-10-421 20003500 PMC2803857

[60] Sugawara N, Wang X, Haber JE. *In vivo* roles of Rad52, Rad54, and Rad55 proteins in Rad51-mediated recombination. Mol Cell. 2003;12(1):209–19. 10.1016/s1097-2765(03)00269-7 12887906

[61] Tsukuda T, Trujillo KM, Martini E, Osley MA. Analysis of chromatin remodeling during formation of a DNA double-strand break at the yeast mating type locus. Methods. 2009;48(1):40–5. 10.1016/j.ymeth.2009.02.007 19245836 PMC2760393

