# Supplementary figures and images for: Interaction of yeast Rad51 and Rad52 relieves Rad52-mediated inhibition of de novo telomere addition
Source: PLoS Genet. 2020 Feb 3;16(2):e1008608. doi: 10.1371/journal.pgen.1008608 (PMC7018233; doi:10.1371/journal.pgen.1008608)

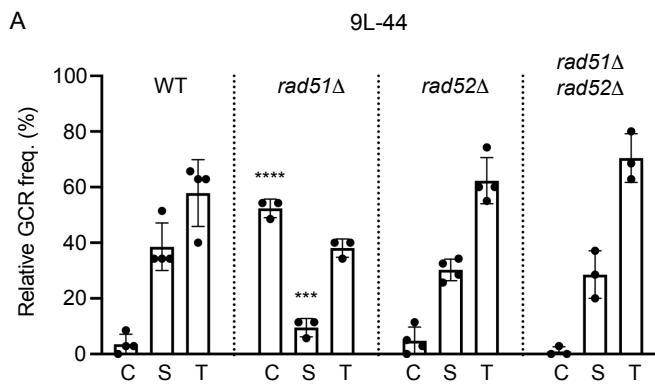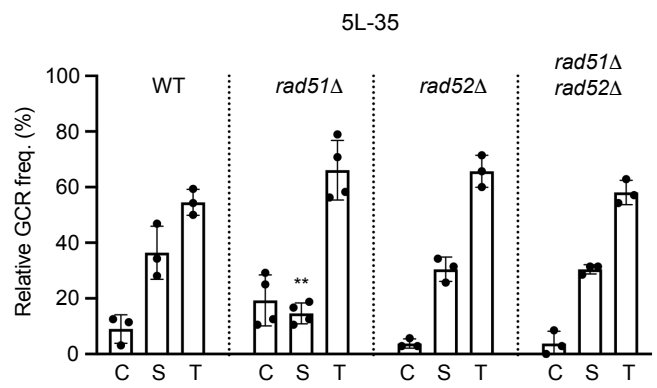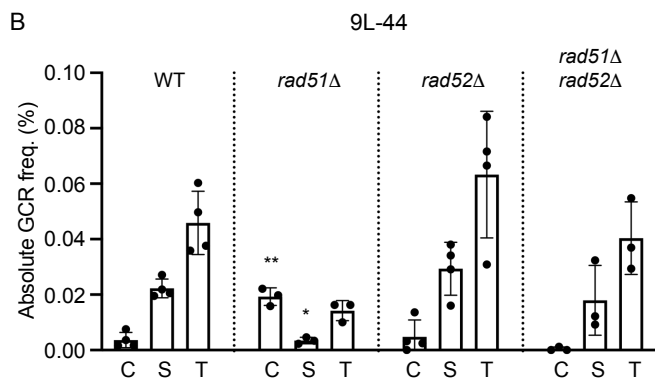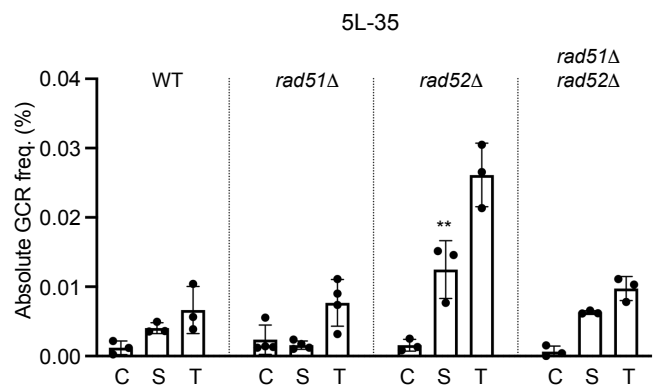

Supplement: S1 Fig — (A) The relative GCR frequency in each region on chromosome 9 (left) or chromosome 5 (right) is shown in the indicated strains. C, S, and T indicate centromere-proximal, SiRTA, and telomere-proximal events, respectively. Data are from the same experiments shown in Figs 1B and 2A. Values are averages of at least three independent experiments with standard deviation. For the centromere-proximal and SiRTA regions only, averages were compared to the WT sample in that same region by ANOVA with Dunnett’s multiple comparisons test. (B) The absolute GCR frequency (see Fig 1C for calculation) in each region on chromosome 9 (left) or 5 (right) is shown in the indicated strains from the same experiments as panel A. Values are averages from three independent experiments with standard deviation. For the centromere-proximal and SiRTA regions only, averages were compared to the WT sample in that same region by ANOVA with Dunnett’s multiple comparisons test (*p<0.05; **p <0.01; ***p<0.001; ****p<0.0001). (PDF) [file pgen.1008608.s001.pdf]

A

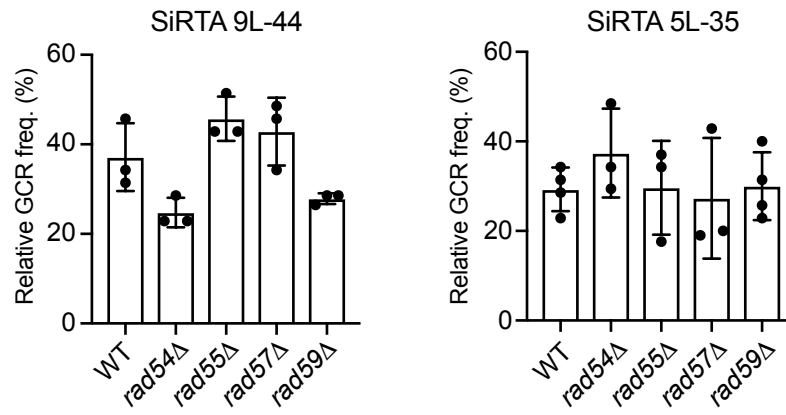

B

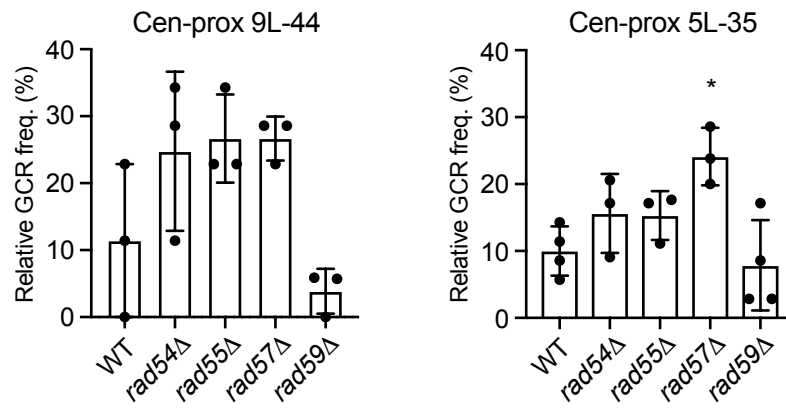

Supplement: S2 Fig — (A) The relative GCR frequency in SiRTA 9L-44 and 5L-35 is shown for the indicated strains. (B) The relative GCR frequency in the region centromere-proximal to SiRTA 9L-44 and 5L-35 is shown for the same experiments in panel A. Averages of at least three independent experiments are shown with standard deviation. Values statistically different from WT by ANOVA with Dunnett’s multiple comparisons test are indicated by asterisks (*p <0.05). Overall GCR frequencies of the strains analyzed in this figure did not differ significantly from those measured in the RAD51 and rad51Δ strains within the same chromosome region. (PDF) [file pgen.1008608.s002.pdf]

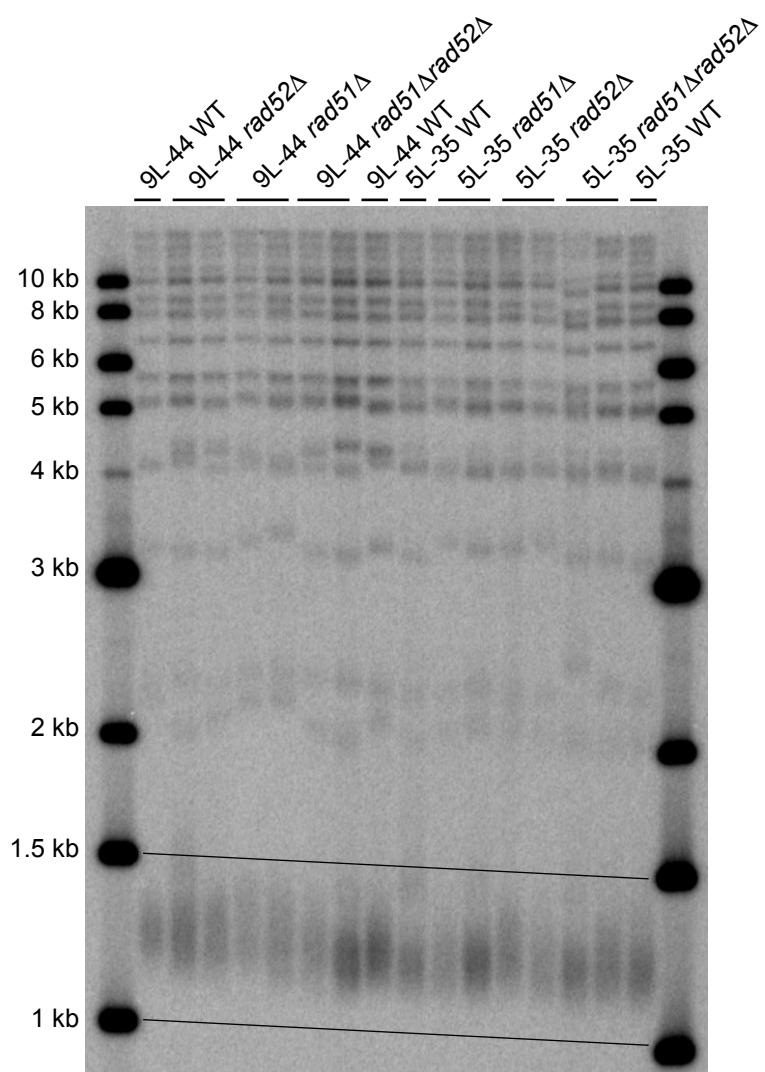

Supplement: S3 Fig — Southern blot analysis endogenous telomeres in WT, rad51Δ, rad52Δ, and rad51Δ rad52Δ strains. 9L-44 and 5L-35 indicate the YKF1752 and YKF1342 strain backgrounds, respectively (S2 Table). The first and last lanes contain molecular weight marker as indicated. (PDF) [file pgen.1008608.s003.pdf]

SiRTA probe

1 2 3 4 5 6 7 8 9 10 11 12 13 14 15 16 17 18 19 20

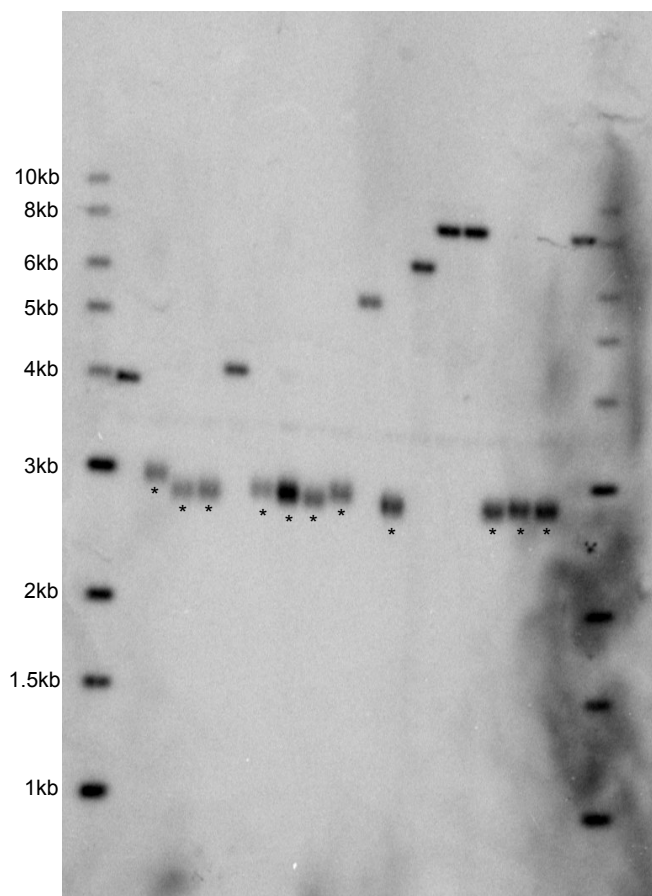

Telomere probe

1 2 3 4 5 6 7 8 9 10 11 12 13 14 15 16 17 18 19 20

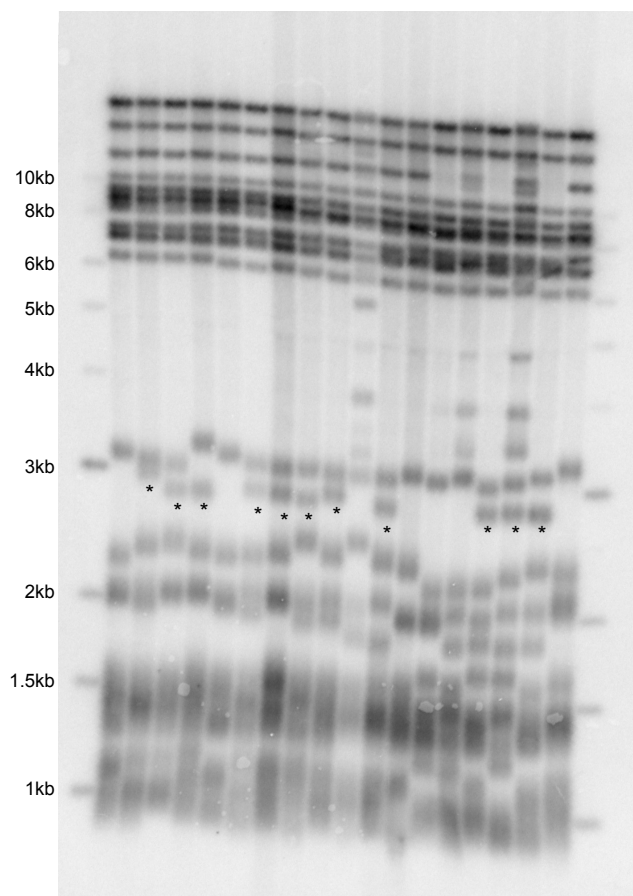

Supplement: S4 Fig — (A) Diagram of the region of chromosome 9 surrounding SiRTA 9L-44 in a WT strain (top) or a strain that has undergone de novo telomere addition at SiRTA 9L-44 (bottom). Sites of cleavage by NsiI and the probes utilized in panel B (SiRTA probe) and C (telomere probe) are shown. (B) A Southern blot conducted on 17 independent GCR events that mapped to SiRTA 9L-44 by PCR (lanes 2–18) was probed with a PCR product located immediately centromere-proximal to SiRTA 9L-44 (see panel A). Lane 19 contains DNA isolated from a WT strain before HO cleavage. Lanes 1 and 20 contain molecular weight marker as indicated. Telomere addition events are indicated with asterisks (*). (C) The same blot shown in B was stripped and reprobed with a short fragment of yeast telomeric DNA. Although multiple bands are detected, the same fragments indicated in panel B are evident (*). (PDF) [file pgen.1008608.s004.pdf]

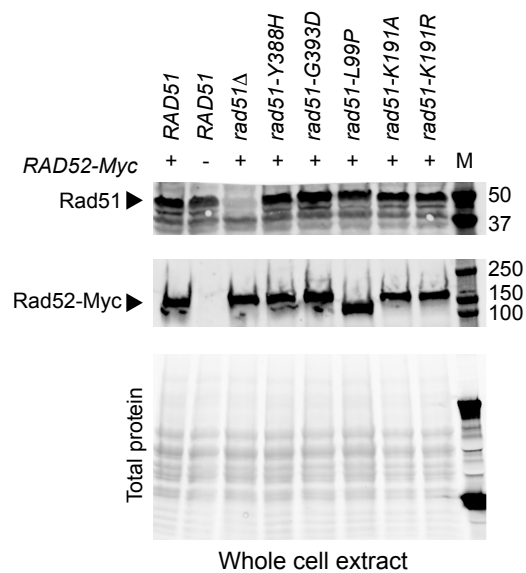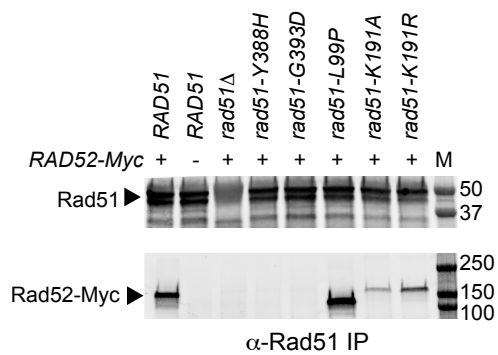

Supplement: S5 Fig — Whole cell protein extracts were generated from strains expressing the indicated RAD51 alleles. Strains contained Myc-tagged RAD52 with the exception of the strain in lane 2. Left panel: Whole cell extracts were probed with anti-Rad51 (top) or anti-Myc (middle) antibodies. Prior to blotting, total protein load was assessed (bottom). Right panel: The same extracts were immunoprecipitated using the anti-Rad51 antibody and probed for Rad51 (top) or Myc (bottom). Sizes of molecular weight markers are indicated (kilodaltons). The L99P strain contains fewer Myc epitopes than the other strains as determined by PCR of the genomic DNA, resulting in slightly faster migration of the Rad52-Myc protein. (PDF) [file pgen.1008608.s005.pdf]
