# Supplementary material for: Interaction of yeast Rad51 and Rad52 relieves Rad52-mediated inhibition of de novo telomere addition
Source: PLoS Genet. 2020 Feb 3;16(2):e1008608. doi: 10.1371/journal.pgen.1008608 (PMC7018233; doi:10.1371/journal.pgen.1008608)
Supplement: S1 Data — (PDF) [file pgen.1008608.s006.pdf]

v.

S1 Data. Summary of nanopore sequencing results for 12 independent Gal^R 5-FOA^R colonies in the *rad51Δ* background. See Fig 3 for the specific locations of breakpoints. Roman numerals below refer to the events illustrated in Fig 3. Because there was insufficient sequence depth to assemble the entire genome, we have not attempted to verify whether each breakpoint represents a simple rearrangement (*i.e.* there could be additional chromosome rearrangements distant to the HO cleavage site that were not detected).

The sequencing data from this study have been submitted to the NCBI Sequence Read Archive (SRA) under BioProject accession number PRJNA557764. The names indicated below correspond to the submitted file names.

GCR01	Chromosome 9 deletion (iv)*
GCR02	5-9 translocation (i)
GCR03	Chromosome 9 deletion (iv)
GCR04	Chromosome 9 deletion (iv)*
GCR05	Chromosome 9 deletion (iv)
GCR06	11-9 translocation (ii)
GCR07	Chromosome 9 deletion (iv)
GCR08	Chromosome 9 deletion (iv)
GCR09	Chromosome 9 deletion (iv)*
GCR10	Chromosome 9 deletion (v)
GCR11	14-9 translocation (iii)
GCR12	Chromosome 9 deletion (iv)*

*As described in the text, the terminal >15kb of chromosome 9 and 10 are nearly identical. In the indicated samples, sequences read(s) including the breakpoint did not extend far enough to include a polymorphism that allows an internal deletion on chromosome 9 to be distinguished from a 10-9 translocation.

Oligonucleotide primers used for PCR verification of breakpoints:

i.	GGTAAAATGATAGCGTCCGTTG
	GAGCAACTCGACGGAGGCTAC
ii.	CTTGATGATTGAACTCATCGCAC
	GTCGTAATGCTGATGCTGGTGC**
iii.	GCCTCAAAGTGGTGAAACTTC
	GTCGTAATGCTGATGCTGGTGC**
iv.	CTTTTACAGAAATGCTTGATAATGC
	CTGCTACAAGACTAAATACGTAC
v.	CCGTGAATAACAACACGATCC
	CTAACTTAACTCTTTTCTCAGTAG

**These primers are identical since the translocations share a breakpoint on chromosome 9.
