# Supplementary material for: Interaction of yeast Rad51 and Rad52 relieves Rad52-mediated inhibition of de novo telomere addition
Source: PLoS Genet. 2020 Feb 3;16(2):e1008608. doi: 10.1371/journal.pgen.1008608 (PMC7018233; doi:10.1371/journal.pgen.1008608)
Supplement: S1 Table — (PDF) [file pgen.1008608.s008.pdf]

S1 Table: Percent telomere addition at SiRTA

STRAIN	TELOMERE ADDITION
9L-44 WT	46/50 (92%)
9L-44 *rad51Δ*	10/19 (53%)
9L-44 *rad52Δ*	39/41 (95%)
9L-44 *rad51Δrad52Δ*	29/29 (100%)
9L-44 *rad51-K191A*	25/25 (100%)
9L-44 *rad51-K191R*	19/25 (76%)
9L-44 *rad51-L99P*	24/25 (96%)
9L-44 *rad52Δ409-12*	12/16 (75%)
9L-44 *rad51Δ rad52Δ409-12*	4/12 (33%)
9L-44 *rfa1-44*	20/27 (74%)
9L-44 *rad51Δ rfa1-44*	24/29 (83%)
5L-35 WT	27/28 (96%)
5L-35 *rad51Δ*	22/23 (95%)
5L-35 2xUAS GBD (*RAD51*)	3/3 (100%)
5L-35 2xUAS GBD-CDC13 (*RAD51*)	19/19 (100%)
5L-35 2xUAS GBD (*rad51Δ*)	4/4 (100%)
5L-35 2xUAS GBD-CDC13 (*rad51Δ*)	20/21 (95%)
5L-35 *rfa1-44*	31/32 (97%)
5L-35 *rad51Δrfa1-44*	24/24 (100%)
