# Supplementary material for: Interaction of yeast Rad51 and Rad52 relieves Rad52-mediated inhibition of de novo telomere addition
Source: PLoS Genet. 2020 Feb 3;16(2):e1008608. doi: 10.1371/journal.pgen.1008608 (PMC7018233; doi:10.1371/journal.pgen.1008608)
Supplement: S2 Table — (PDF) [file pgen.1008608.s009.pdf]

S2 Table. List of strains.

Strain	Genotype	Source (ref)
YKF1308	JRL017: *MAT**a**::ΔHOcs::hisG hmlαΔ::hisG HMRa-stk ura3Δ851 trp1Δ63 leu2Δ::KAN*^{R} *ade3::GAL10::HO can1,1-1446::HOcs::HPH*^{R}	(29)
YKF1310	CL11-7: *MAT**a**::ΔHOcs::hisG hmlαΔ::hisG HMRa-stk ura3Δ851 trp1Δ63 leu2Δ::KAN*^{R} *ade3::GAL10::HO*	(29)
YKF1333	YKF1310 *hmra-stkΔ::NAT*^{R}	(20)
YKF1323	YKF1308 *hmra-stkΔ::NAT*^{R}	(20)
YKF1342	YKF1323 *hxt13*::*URA3* (WT 5L-35 strain)	(20)
YKF1752	YKF1333 Chr9;35050-41450::*HOcs::HPH*^{R} *soa1*::*URA3* (WT 9L-44 strain)	This study
YKF1784	YKF1752 *rad51::TRP1*	This study
YKF1885	YKF1752 *rad51::LEU2*	This study
YKF1785	YKF1752 *rad52::LEU2*	This study
YKF1791	YKF1784 *rad52::LEU2*	This study
YKF1718	YKF1342 *rad51::TRP1*	This study
YKF1783	YKF1342 *rad52::LEU2*	This study
YKF1835	YKF1718 *rad52::LEU2*	This study
YKF1811	YKF1752 *rad54::TRP1*	This study
YKF1841	YKF1752 *rad55::TRP1*	This study
YKF1842	YKF1752 *rad57::TRP1*	This study
YKF1843	YKF1752 *rad59::TRP1*	This study

YKF1867	YKF1885 *rad59::TRP1*	This study
YKF1815	YKF1811 *rad51::LEU2*	This study
YKF1821	YKF1342 *rad54::TRP1*	This study
YKF1822	YKF1342 *rad55::TRP1*	This study
YKF1824	YKF1342 *rad57::TRP1*	This study
YKF1826	YKF1342 *rad59::TRP1*	This study
YKF1921	YKF1752 SiRTA 9L-44Δ	This study
YKF1923	YKF1921 *rad51::LEU2*	This study
YKF 1986	YKF1752 *rad51-Y388H*	This study
YKF1984	YKF1752 *rad51-G393D*	This study
YKF1987	YKF1752 *rad51-L99P*	This study
YKF1989	YKF1752 *CDC13-13Myc-TRP1*	This study
YKF1990	YKF1885 *CDC13-13Myc-TRP1*	This study
YKF2048	YKF1989 *rad52::LEU2*	This study
YKF2085	YKF1989 *rad51Δ rad52::LEU2*	This study
YKF 2074	YKF1752 *rfa1-44*	This study
YKF2076	YKF1784 *rfa1-44*	This study
YKF2078	YFK1342 *rfa1-44*	This study
YKF2088	YKF1718 *rfa1-44*	This study
YKF2082	YKF1752 *rad52Δ409-12*	This study
YKF2084	YKF1784 *rad52Δ409-12*	This study

YKF2038	YKF1752 *pol32::TRP1*	This study
YKF2040	YKF1885 *pol32::TRP1*	This study
YKF2085	YKF1989 *rad51Δ rad52::LEU2*	This study
YKF2109	YKF1752 *rad51-K191R*	This study
YKF2111	YKF1752 *RAD52-13Myc-TRP1*	This study
YKF2112	YKF1986 *RAD52-13Myc-TRP1*	This study
YKF2113	YKF1984 *RAD52-13Myc-TRP1*	This study
YKF2114	YKF1885 *RAD52-13Myc-TRP1*	This study
YKF2115	YKF1987 *RAD52-13Myc-TRP1*	This study
YKF2117	YKF2107 *RAD52-13Myc-TRP1*	This study
YKF2118	YKF2109 *RAD52-13Myc-TRP1*	This study
YKF1588	*hmra::NAT* 5L-35 *HS::2XUAS hxt13::URA3*	(20)
YKF1788	YKF1588 *rad51::TRP1*	This study
