# Supplementary material for: Interaction of yeast Rad51 and Rad52 relieves Rad52-mediated inhibition of de novo telomere addition
Source: PLoS Genet. 2020 Feb 3;16(2):e1008608. doi: 10.1371/journal.pgen.1008608 (PMC7018233; doi:10.1371/journal.pgen.1008608)
Supplement: S3 Table — (PDF) [file pgen.1008608.s010.pdf]

S3 Table. List of primers for chromatin immunoprecipitation

NAME	SEQUENCE	REFERENCE/USE
9L-44 NEW RT FOR 2	AATACAGGAAACACCATC	ChIP DNA amplification for 9L-44 locus
HS CHECK PSiRTA 2 REV	CTAGGAACACAACCTAATTACC	Used with 9L-44 NEW RT FOR 2
ARO1 FOR	TCGTTACAAGGTGATG	ChIP control DNA amplification (63)
ARO1 REV	AATAGCGGCAACAAC	Used with ARO1 FOR
B4 BNR1 FOR	GGAAGGATCTCTGACCATCCC	Probe for *de novo* telomere addition
B4 BNR1 REV	GAACGCATGCTATGCTGAACG	Used with BNR1 FOR
